# Supplementary material for: Chromothripsis-associated chromosome 21 amplification orchestrates transformation to blast-phase MPN through targetable overexpression of DYRK1A
Source: Nat Genet. 2025 Jun 9;57(6):1478–92. doi: 10.1038/s41588-025-02190-6 (PMC12165854; doi:10.1038/s41588-025-02190-6)
Supplement: Supplementary file 1 — Supplementary Note, Methods and References. [file 41588_2025_2190_MOESM1_ESM.pdf]

nature genetics

Supplementary information

https://doi.org/10.1038/s41588-025-02190-6

Chromothripsis-associated chromosome 21 amplification orchestrates transformation to blast-phase MPN through targetable overexpression of *DYRK1A*

In the format provided by the authors and unedited

Supplemental note: Chromothripsis-associated chromosome 21 amplification orchestrates transformation to blast phase MPN through targetable overexpression of *DYRK1A*

Intrachromosomal amplification of chromosome 21 (iAMP21) is well-described in pediatric B-cell precursor ALL (B-ALL). In this context, recurrent breakage-fusion-breakage cycles lead to the amplification of chr21q in ~2% of B-ALL patients, an event associated with high-risk disease.[1–3] The consensus chromothripsis landscape in iAMP21 also mirrors the CNA landscape across large cohorts of cancer samples, supporting a pivotal role of chromothripsis in fine tuning the CN landscape of chromosome 21.[2] The chr21amp event we describe in BP-MPN shares some similarities with iAMP21 in B-ALL. A recent study of 124 iAMP21 pediatric ALL cases identified that iAMP21 is an early, clonal event, typically with breakage-fusion-bridge cycles as the initiating event, often followed by chromothripsis.[4] In our BP-MPN cohort the boundaries of the amplified regions are similarly frequently demarcated by fold-back inversion rearrangements indicative of breakage-fusion-bridge cycles.[4,5] The MAR identified in iAMP21-ALL aligns closely with the smaller MAR seen in BP-MPN, with *DYRK1A* at its center, with transcriptional upregulation of *DYRK1A* also observed in iAMP21-ALL.[4] However, there are also important differences between iAMP21-ALL and chr21amp in BP-MPN. In the MPN context, chr21amp arises in HSC as opposed to B-cell progenitors and also occurs as a late event immediately prior to leukemic transformation, in the context of an antecedent MPN clone.

DYRK1A has been studied in a range of disease contexts, most closely in Down syndrome (DS) where congenital *DYRK1A* upregulation occurs by trisomy and has been implicated in the cardiac, hematological and neurological features of DS.[6–9] Further diverse roles have been proposed for DYRK1A, including the phosphorylation of tau and amyloid proteins and in regulating pancreatic beta cell proliferation, leading to its investigation as a modulator of diseases ranging from Alzheimer's to diabetes.[7,10–12] Consequently, several DYRK1A inhibitors have been developed which show on-target efficacy and negligible toxicity in murine models and humans.[13–15] Inhibition of DYRK1A has also recently been reported to alter splicing and increase sensitivity to BCL2 inhibition in non-*DYRK1A* amplified AML.[16]

Here, we propose that two biological pathways activated by *DYRK1A* are crucial in its oncogenic activity in BP-MPN. First, we suggest that the chr21amp event causes downregulation of DNA repair pathways to promote genomic instability. A recent study in the context of Down Syndrome associated myeloid malignancies demonstrated that increased expression of *DYRK1A*, which lies in the centre of the Down Syndrome critical region, leads to impaired homology-directed DNA repair as a mechanism of elevated mutagenesis, providing additional support for our proposed model in BP-MPN.[17,18] This is also in accordance with molecular events in Fanconi Anemia, where it is established that congenital loss of the Fanconi DNA repair pathway followed by *TP53* downregulation drives a survival advantage and stem/progenitor cell survival in leukemia development.[19,20] Notably, these events occur in a different order to our observations in BP-MPN, where *TP53* mutation precedes *DYRK1A* mediated repression of DNA repair pathways.

Second, we demonstrate that *DYRK1A* upregulation leads to amplification of *JAK2V617F*-driven upregulation of JAK/STAT signalling in BP-MPN, with clear parallels to the B-cell ALL iAMP21 clinical subgroup, which is characterized by an enrichment in JAK/STAT signalling mutations.[2–4,21] Our interrogation of published *de novo* AML datasets for the chr21amp event confirms its co-occurrence with *TP53* and rarity outwith the JAK/STAT mutant setting, corroborating a small case series of chr21amp AML patients.[22] Furthermore, myeloid leukemia in Down Syndrome shows a very high prevalence (48%) of mutations leading to activation of JAK family kinases as well as increased chromosomal CNAs, a finding recently linked to *DYRK1A* overexpression.[17,23] We herein provide experimental evidence that the association between *DYRK1A* amplification and JAK/STAT mutation likely relates to enhanced JAK/STAT signalling mediated by *DYRK1A* overexpression. This further potentiation of an oncogenic pathway already activated by mutation by CNAs is documented in other cancer settings, such as in the case of *BRAF* mutant solid tumors.[24] We propose chr21amp as a biomarker of adverse clinical outcome in BP-MPN, where it synergizes with JAK/STAT pathway mutations and represents an example of “aneuploidy addiction”, akin to an oncogene addiction. This biological concept is supported by recent data demonstrating that genetically engineered loss of aneuploidy, in this instance generating disomic lines from trisomic 1q+ cancer cell lines, abrogates their oncogenic potential.[24,25]

Supplementary Methods

Single Nucleotide Polymorphism Array Copy Number Variant and Loss of Heterozygosity Analysis

To call mosaic copy number events in primary patient samples, genotyping intensity data generated was analysed using the Illumina Infinium OmniExpress v1.3 BeadChips platform. Haplotype phasing, calculation of log R ratio (LRR) and B-allele frequency (BAF) and calling of mosaic events was performed using Mocha (Mocha: A BCFtools extension to call mosaic chromosomal alterations starting from phased VCF files with either B Allele Frequency (BAF) and Log R Ratio (LRR) or allelic depth (AD)).[26,27] In brief, Mocha comprizes the following steps: (1) filtering of constitutional duplications; (2) use of a parameterized hidden Markov model to evaluate the phased BAF for variants on a per-chromosome basis; (3) deploying a likelihood ratio test to call events; (4) defining event boundaries; (5) calling copy number; (6) estimating the cell fraction of mosaic events. A series of stringent filtering steps was applied to reduce the rate of false positive calls. To eliminate possible constitutional and germline duplications, excluding calls with lod_baf_phase <10, those with length <500kbp and rel_cov>2.5, and any gains with estimated cell fraction >80%, logR>0.5 or length <24Mb. Given that interstitial LOH are rare and likely artefactual, all LOH events <8Mb were filtered.[26,27] Events on genomic regions reported to be prone to recurrent artefact (chr6<58Mb, chr7>61Mb, and chr2 >50Mb) were also filtered, and those where manual inspection demonstrated noise or sparsity in the array.

To find common genomic lesions on a focal and arm level, Infinium OmniExpress arrays were initially processed with Illumina Genome Studio v2.0.4. Following this, Log R Ratio (LRR) data was extracted for all probes and array annotation obtained from Illumina (InfiniumOmniExpress-24v1-3_A1). LRR data was then smoothed and segmentation called using the CBS algorithm from the DNACopy v1.60.0 package in R.[28,29] A minimum number of 5 probes was required to call a segment, and segments were analysed using GenomicRanges v1.38.0.[30,31] Definitions of amplification, gain, loss and deletion events are outlined in Bashton, *et al.* Segmentation data was further analysed in GISTIC v2.023.[32] Co-occurrence of CNAs and mutation status was calculated by Pearson correlation coefficient as implemented in the R package *corrplot* (v0.84). Chromothripsis was defined in cases

meeting two standards (1) according to Korbel and Campbell's criteria when three out of six criteria (those assessable by SNP array analyses) were satisfied, and (2) according to the criteria by Rausch et al, requiring 10 changes in segmental copy number involving 2 or 3 distinct copy number states on a single chromosome.[33,34]

Analysis of external AML datasets

Data retrieval and pre-processing

Three publicly available AML cohorts with genetic mutation +/- copy number profiling +/- RNA-sequencing data available were used to validate findings from our single-cell analysis, namely BeatAML[35], The Cancer Genome Atlas (TCGA)[36] and a large *de novo* AML dataset from Tazi *et al*.[37] For BeatAML and TCGA datasets, gene expression values in FPKM (fragments per kilobase of transcript per million mapped reads) were retrieved from the National Cancer Institute (NIH) Genomic Data Commons (GDC).[38] Gene expression values were then offset by 1 and log2-transformed. *TP53* point mutation status was retrieved from the cBio Cancer Genomics Portal (cBioPortal).[39] Clinical data including survival data for BeatAML and TCGA was retrieved from the BeatAML data viewer (Vizome) and NIH GDC, respectively.

We selected samples from the BeatAML cohort with an AML diagnosis collected within 1 month of the patient's enrolment in the study, with RNA-sequencing and survival data available (360 *de novo* AML in total). The TCGA cohort consists of 200 *de novo* AML patients represented by one sample each, out of which 191 patients had copy number and survival data (9 chr21amp, 182 non-chr21amp), 119 had *mTP53* status (108 *TP53*-WT and 11 *TP53*-mutant) and 127 had concomitant RNA-sequencing data available.

Patients were first split by copy number status over chromosome 21. This resulted in two groups of patients, namely patients with high copy number (greater than or equal to 2.5) over the minimally amplified region on chr21, and patients with normal/low copy number (<2.5). Co-occurrence of mutation status and chr21amp was assessed using either the Chi-square or Fisher's exact test.

Survival analyses

Overall survival was calculated according to the method of Kaplan and Meier, and a Cox proportional hazards regression model was fitted to estimate the hazard ratio, as implemented in the R package *survival* (v3.2-10). The log-rank test was performed to identify differences between curves. Kaplan-Meier curves were plotted using the *survminer* (v0.4.9) R package to visualize the probability of survival and sample size at a respective time interval. Multivariate analysis was performed using a Cox proportional hazards model to evaluate the effects of covariates on outcome, using the backwards Wald test to assess significance and implementing *mTP53* status as an interaction term. Multivariable analysis was performed in SPSS (v29.0.0)

Whole genome sequencing analysis

Unmatched tumor-only whole genome sequencing analysis was performed using the commercial Isabl platform pipeline and interface.[40] All bioinformatic tools were launched using an in-house wrapper.

Whole genome paired-end reads were aligned to human reference genome (GRCh37d5) using BWA-mem (v0.7.17) as a part of the pcap-core v2.18.2 wrapper (https://github.com/cancerit/PCAP-core).[41] The wrapper includes marking of duplicates using Picard. Mosdepth 4 was deployed to calculate genome-wide median coverage.[42] cgpBattenberg (v1.4.0) was used to estimate tumor purity/ploidy and allele-specific subclonal copy number changes (https://github.com/cancerit/cgpBattenberg).[43] Single nucleotide variants (SNVs) were identified using Strelka2 (v2.9.1 with manta v1.3.1), (https://github.com/Illumina/strelka), MuTect2 (gatk:v4.0.1.2), (https://github.com/broadinstitute/gatk) and CaVEMan (cgpCavemanWrapper v1.7.5) (https://github.com/cancerit/cgpCaVEManWrapper).[44–46]

Variant post-processing was done using default flags for Strelka2 and MuTect2 while for CaVEMan, cgpCavemanPostprocessing (v1.5.2) was used filtering for sequencing artefacts utilizing a panel of 100 unmatched normals (https://github.com/cancerit/cgpCaVEManPostProcessing). Small insertions and deletions (indels) were detected using Strelka2, MuTect2, and Pindel (cgpPindel v1.5.4) (https://github.com/cancerit/cgpPindel) and filtered against a panel of unmatched normals.[47] SvABA (~v1.0.0 commit 47c7a88) (https://github.com/walaj/svaba), GRIDSS (v2.2.2)

(https://github.com/PapenfussLab/gridss) and BRASS (v4.0.5 with GRASS v1.1.6) (https://github.com/cancerit/BRASS) were used to call structural variants against a panel of unmatched normals.[48,49] Merged VCFs were annotated with VAGrENT(v3.3.0, https://github.com/cancerit/VAGrENT) and VEP (v92, https://github.com/Ensembl/ensembl-vep).[50,51] High-confidence substitutions and indels were designated as those that were passed by at least 2 callers. Variants were further filtered to exclude those present in a panel of 100 unmatched normals or in any of the germline variation databases in VEP.

Chromosome 21 amplification timing analysis

Timing analyses were restricted to segments with three or more SNVs passed by two or more variant callers. The multiplicity of each variant was derived from the variant allele frequency provided by Mutect2, or Strelka in cases where a variant was not passed by Mutect2, using version 1.0.8 of the dpclust3p R package.[52,53] These data were combined with Battenberg copy number calls to calculate timing of the amplification with all available mutations using the AmplificationTimeR R package.[54] The algorithm uses copy number states in combination with information about the multiplicity of mutations within the copy number altered segment to work out the timing of individual gains in pseudotime. In the simplest scenario possible where only one chromosome copy is gained resulting in a copy number state of 2+1 (major allele + minor allele), mutations occurring on the major allele prior to the gain will be present on two chromosomes (multiplicity 2) at the time of sampling. Mutations occurring after the gain on the major allele, or at any time during the history of the tumour on the minor allele, will be present on only one chromosome (multiplicity 1). If one assumes that mutation rate is constant, one can tally the number of mutations at multiplicity 1 (n_1) and multiplicity 2 (n_2) and use these to calculate the time of the gain. This logic can be extended to higher copy number gains with higher multiplicity states, with the highest possible multiplicity state corresponding to the copy number of the major allele (n_{Maj}).

Breakpoint analysis

Breakpoint junctions and associated discordant read pairs and soft-clipped reads were identified and classified as in Cortes-Ciriano et al[55] (originally adapted from Kidd *et*

al[56]), whereby the sequence was interrogated for features of transposable element insertion (TEI), variable number of tandem repeats (VNTR), nonhomologous end joining (NHEJ), alternative end joining (alt-EJ), nonallelic homologous recombination (NAHR), and fork stalling and template switching/microhomology-mediated break induced repair (FoSTeS/MMBIR). A median of 5 (range 5-11) chromosome 21 breakpoints were examined per case.

ecDNA enrichment and sequencing for ecDNA detection

High molecular weight genomic DNA was isolated from the primary patient sample using the Qiagen MagAttract HMW DNA kit (Cat. No./ID: 67563) and amplified using the Qiagen REPLI-g Mini Kit (Cat. No. / ID: 150025). Amplified DNA was subjected to T7 endonuc lease digestion to reduce DNA branching, using the Endo-T7 Nuclease from New England Biolabs (M0302S). The amplified and T7 endonuclease digested DNA was cleaned up with AMPure XP Beads (Beckman Coulter A63881). The library was prepared using the Ligation Sequencing Kit SQK-LSK110 from Oxford Nanopore Technologies Ltd, Oxford, UK, according to the manufacturer’s instructions. The library was sequenced on a MinION using R9.4.1 Flowcell (FLO-MIN106, Oxford Nanopore Technologies Ltd, Oxford, UK) for more than 47 h. The raw FAST5 sequencing data was basecalled using Guppy (version 6.4.6). Reads were quality-filtered using NanoFilt[57](2.8.0) and aligned using ngmlr[58](version 0.2.7) against the GRCh38/hg38 reference genome (https://github.com/henssen-lab/nano-wgs). For ecDNA detection and reconstruction Decoil[59](version 1.1.2) was applied using the decoil-pipeline in sv-reconstruct mode, which calls SVs using Sniffles (version 1.0.12).[58]

TARGET-seq

Individual HSPCs were isolated by index flow cytometry, enriching for early stem cell populations (Lin-CD34+CD38- CD45RA-CD90+) followed by integrated single cell genotyping at allelic resolution for m*TP53* and *JAK2* and single cell RNA sequencing (sc-RNA-seq), leveraging the softwares *inferCNV* and *numbat* to call CNAs in single cells.[60–62] The processed count matrix for 8 myelofibrosis (MF) patients,14 BP-MPN patients and 9 healthy donors profiled using TARGET-seq were downloaded from GSE226340, normalized by library size and log2-transformed.[62] HSPCs were classified as MF (MF non-TP53 mutant controls) chr21amp_TP53_MT (chr21amp

tp53 mutant cells), TP53_MT _no_chr21amp (non-chr21amp, tp53 mutant cells), pre-LSC (WT cells from BP-MPN donors) or WT-normal (cells from normal donors). Differentially expressed genes were identified using a combination of the non-parametric Wilcoxon test, to compare the expression values for each group, and Fisher's exact test, to compare the frequency of expression for each group.[63] p-values were combined using Fisher's method, and adjusted p-values derived using the Benjamini & Hochberg procedure. Significant genes were selected on the basis of a log2(fold change)>1 and adjusted p value < 0.05.[64,65]

Bulk RNA-seq analysis

Data pre-processing

Illumina sequencing data in the binary base call (BCL) format was demultiplexed using bcl2fastq v2.20.0.422. Data quality was assessed using FastQC v0.11.5 (https://github.com/s-andrews/FastQC). Nextera adapters and 3' bases with Phred quality score less than 20 were trimmed from the single-end reads using Trim Galore v0.6.5 (https://github.com/FelixKrueger/TrimGalore). Trimmed reads were subsequently mapped to the hg19 human reference genome using STAR v2.6.1d in 2-pass mode.[66]

Gene expression quantification

Mapped reads were quantified using featureCounts (part of the Subread v2.0.0 package suite)[67], and the gene expression counts were summarized by gene identifiers based on GENCODE V10. For each sample, the raw counts were normalized by the corresponding sample's library size (total raw counts) and then multiplied by 1,000,000 to obtain the gene expression values in counts per million (CPM) unit.

Differential gene expression analysis

Differential gene expression was carried out with the *DESeq2* R package (v1.28.1).[68] Differentially expressed genes were identified using Wald test pairwise analysis and *P* values were adjusted for multiple testing using the Benjamini and Hochberg method. Genes were filtered to those expressed (defined as log2(CPM)>1) by a minimum of 3 samples in each group. Unless otherwise specified, adjusted *p* values < 0.05 and log2-fold change 1 and -1 cut-offs were applied to define

significantly up- or down-regulated genes, respectively. Heatmaps were generated using the package *ComplexHeatmap* R package (v 2.14.0) and volcanos were plotted using *EnhancedVolcano* R package (v1.16.0).

Gene set enrichment analysis

GSEA was performed using the GSEA software (Broad Institute; v4.3.2, RRID: SCR_003199) inputting the normalized count matrix and incorporating expressed genes as a background gene list, against Hallmark (h.all.v2023.1) and curated KEGG gene sets (c2.cp.kegg.v2023.1), obtained through the GSEA GUI (Broad Institute; RRID: SCR_003199) using the default settings.[69,70]

Allele specific gene expression analysis

The whole genome sequencing VCF files were filtered to identify informative heterozygous SNPs located in the chr21amp genes with coverage from the Smart-seq2 RNA-seq data (exonic or 3'UTR) for each case (Supplementary Table 4). Parental haplotypes were assigned for each SNP based on VAF. A custom genome file with replacement of REF alleles by ALT was generated prior to re-aligning the trimmed RNA-seq FASTQs to this custom genome file to enable alignment correction. Read-counting was performed for both the WT and ALT aligned BAM files to enable maximal capture of all reads aligning to the SNP site, and read counting was performed to assess for allelic skew.

Bulk ATAC-seq analysis

Data pre-processing

Illumina sequencing data in the binary base call (BCL) format were demultiplexed. using bcl2fastq v2.20.0.422. Adapter trimming was performed using Trim Galore v0.6.5. Trimmed reads were aligned to the hg19 human reference genome with Bowtie2 v2.4.2.[71] Duplicate reads were removed using *MarkDuplicates* module from Picard v2.3.2 and mitochondrial reads were removed using Samtools v1.9.[72] ATAC-seq QC was performed using the *fragSizeDist* function of the *ATACseqQC* R package (v1.14.4) to assess for fragment size distribution whereas mapped, mitochondrial and duplicated reads were calculated using Samtools.[72] The *shiftGAlignmentsList* function from *ATACseqQC* was further used to shift the

coordinates of aligned reads in the BAM file to account for the 5’ overhang of 9 base long created by the tagmentation of Tn5 transposases. Specifically, coordinates of reads mapping to the positive and negative strand were shifted by +4 and -5, respectively. The resulting BAM file was converted to bigWig format using deepTools and viewed in the UCSC data hub.[73]

<u>Peak calling, annotation, and quantification</u>

BAM files from each genotype were merged and peak calling was performed using MACS2 v2.2.7.1.[74] Each peak was standardized to a fixed width of 500bp.[75] Specifically, for each peak, 250bp was subtracted from and added to the coordinate of the peak summit to obtain the start and end coordinate of the peak, respectively. Next, for each sample, the peaks were ranked according to the their -log10(*p* value). Overlapping peaks were subsequently merged by retaining the peak coordinate with the highest -log10(*p* value). This process was repeated across all samples to generate a consensus peak list for the entire dataset for downstream peak annotation and quantification.

Peak annotation was performed with the *ChIPseeke*r R package (v1.34.1), and *TxDb.Hsapiens.UCSC.hg.knownGene* (v3.2.2) annotation R package. Specifically, each peak was annotated with its nearest gene and whether it is located within the promoter or putative distal regulatory element. The former is defined as located within 1000bp upstream and 100bp downstream of a transcription start site (TSS) while the latter is defined as located outside promoter region.

Peak quantification was performed by counting the number of sequencing reads that align to each peak using the *countOverlaps* function from the *GenomicRanges* (v1.50.2) R package. The raw peak count data was tabulated as a matrix whereby the column represent the peak coordinates, rows represent the sample, and values represent the raw read count. For each sample, the raw counts were normalized by the corresponding sample’s library size (total raw counts) and then multiplied by 1,000,000 to obtain the peak expression values in counts per million (CPM) unit.

<u>Differential peak analysis</u>

Differential peak analysis was carried out in *DESeq2* R package (v1.28.1).[68] Differentially expressed peaks were identified using the Wald test and *P* values were

adjusted for multiple testing using Benjamini-Hochberg. Unless otherwise specified, adjusted *p*-values < 0.05 and log2-fold change 1 and -1 cut-offs were applied to define significantly up- or down-regulated genes, respectively.

<u>Transcription factor motif analysis</u>

Motif calling and discovery analysis was performed using the findMotifsGenome.pl function in Homer v20201202 [76] using both MACS2 narrowPeak and MACS2 summit files extended +/- 200bp as input files and a file of all ATAC peaks as background.

<u>Principal component analysis</u>

Principal component analysis (PCA) was performed using the FactorMineR (v2.8) R package and the eigenvalues for the 1st two principal components were computed using the factoextra (v1.0.7) R package. For PCA of RNAseq, we used highly variable genes as features. Highly variable genes were defined at the top 10% of expressed genes with the highest variance, and these expressed genes were defined as genes with ≥1 CPM in at least 3 samples. For PCA of ATACseq, we used differentially expressed peaks as defined above as features. The gene and peak expression values of RNAseq and ATACseq, respectively, were offset by +1, log2-transformed, and then scaled prior to dimension reduction analysis.

Western blotting

SET2 and HEL cells were treated with DMSO or DYRK1A inhibitor EHT1610 for the time and concentrations indicated in the figure legends. Whole cell lysis was performed with TENT buffer (50mM Tris, pH 8.0, 2mM EDTA, 150mM NaCl, 1% Triton X-100) supplemented with 2mM NaF, 2mM NaVO3, 2mM Sodium Pyrophosphate, 2mM beta-glycerophosphate, and 1x complete protease inhibitor cocktail (Roche) for 30 minutes on ice. Debris was cleared by centrifugation at 21000g for 10 minutes at 4°C. Lysates were denatured in LDS sample loading buffer (Life Technologies) at 95°C for 5 minutes and electrophoresed on 4-15% Mini-Protean TGX gels (BioRad). Proteins were transferred to PVDF membranes (EMD Millipore) and probed with primary antibodies against LIN52, phospho-S28-LIN52[77], FOXO1, phospho-FOXO1 S329, phospho-STAT3 Y705, STAT3, GRB2 and HSC70.

The membranes were then incubated with primary antibodies overnight at 4ºC on a shaking platform. After washing with 1x TBST five minutes for five times, all Western blots were detected by HRP-conjugated secondary antibodies and visualized with SuperSignal™ West Pico PLUS Chemiluminescent Substrate (ThermoFisher Scientific), or were detected with IRDye secondary antibodies (LI-COR) and visualized with Odyssey CLx Imaging System (LI-COR). Primary and secondary antibodies are described in detail in **Supplementary Table 16**. Densitometry values were calculated using ImageJ software (NIH).

High-throughput single-cell RNA-sequencing (10x Chromium)

9,000 CD34+ lineage negative and 9,000 viable mononuclear cells were sorted into 30 μl PBS/0.05% BSA (non-acetylated) in a 1.5ml DNA lo-bind Eppendorf. Two samples with non-overlapping HTOs were hashed, and the cell number/volume adjusted to the target for loading onto the 10x Chromium Controller. Samples were processed according to the 10x protocol using the Chromium Single Cell 30 library and Gel Bead Kits v3.0 (10x Genomics). Cells and reagents were prepared and loaded onto the chip and into the Chromium Controller for droplet generation. Reverse transcription was conducted in the droplets and cDNA recovered through demulsification and bead purification. Pre-amplified cDNA was used for library preparation, multiplexed and sequenced on a Novaseq S4, aiming to obtain > 50,000 reads per cell. A preliminary, low-depth run was performed to more accurately estimate the number of cells and total sequencing required.

10x Genomics single-cell RNA sequencing data pre-processing and integration

Illumina sequencing data in the binary base call (BCL) format were demultiplexed. using bcl2fastq (v2.20.0.422). UMI counts were obtained by aligning FASTQ files to the human reference genome (GRCh38 3.0.0) using Cell Ranger software (v7.0.0) from 10x Genomics. The CellRanger ‘‘count’’ standard pipeline was used to obtain the expression matrix for each individual library for each donor. cite-seq-count/1.4.4 was run using the --expected cells setting for HTO libraries.

Demultiplexing & doublet exclusion

Donors were demultiplexed using the Souporcell pipeline v2.0 and Singularity v3.2. Donor identification was performed using the hashtag oligonucleotide (HTO)

information and the HTODemux() function implemented in Seurat v4. Doublet exclusion was performed using (1) souporcell's incorporation of troublet v2.4 for intergenotypic doublet detection incorporating cluster assignments and cell allele counts and (2) identifying cells labelled by more than one unique HTO. Doublets were excluded from downstream analysis.

Single cell 3'-biased RNA-sequencing data pre-processing

Quality control was performed using the following parameters: number of genes detected > 500, percentage of mitochondrial reads < 15%, percentage of unmapped reads < 75%, min cells expressing genes = 10, minimum UMIs =500. n=6143 HSPCs from healthy donors, n=27549 non-chr21amp BPMPN and n=6572 from chr21amp BPMPN passed QC and were taken forward for analysis (total n= 40264 cells). The median mitochondrial read % of cells passing QC was 3.7% (IQR 2.9-4.7%), median UMI count was 18648 (IQR 12862-26004) and median number of genes detected were 4665 (IQR 3772-5514).

Dimensionality reduction, removal of individual donor effect and cell clustering

To account for any technical batch effects and different sequencing depths, we integrated the healthy control dataset by batch using Seurat integration.[78] After log-normalization of each dataset using `NormalizeData`, we selected the top 2,000 highly variable genes across the datasets using `SelectIntegrationFeatures`, scaled the normalized data and regressed out the percentage of mitochondrial genes. For healthy control samples, we used Reciprocal Principal Component Analysis (RPCA) to integrate across batches using the `FindIntegrationAnchors` function with k=10 and `IntegrateData`, followed by PCA analysis with 30 principal components, and UMAP for Dimension Reduction for dimensionality reduction. Clustering was performed using the Louvain algorithm ("FindClusters'), based on the k-nearest neighbor graph derived from integrated data and using resolution = 1.0.

Marker gene identification and cell type annotation

Differentially expressed genes for each healthy donor cluster were identified using the 'FindAllMarkers' function in Seurat. Differentially expressed genes were identified using the criteria of minimum log2FC 0.5, minimum percent expression 0.1,

and ranked using p values and log2FC to select up to 50 genes per cluster. Clusters were identified by manual inspection of differentially expressed genes for canonical marker genes of blood cell lineages and comparison to reference datasets.[79–82] BP-MPN cells were then reference mapped to healthy control cells using the Seurat *FindTransferAnchors* and *TransferData* reference mapping functions to transfer the cell type annotations. Cells annotated as lymphoid cells were excluded from downstream analyses, leading to a final cell count of 40264 cells.

CNA inference

Inference of chromosomal alterations from single cell gene expression data was performed using a haplotype-aware caller, numbat.[61] Healthy control samples without copy number alterations were used as the expression reference (lambdas_ref). Numbat was run with default parameters. The clustered clonal outputs were reviewed and Numbat output clones (bulk_clones) were assigned to single cells.

Single-cell regulatory network inference and clustering (SCENIC) analysis

SCENIC permits identification of regulons (genes co-expressed with transcription factors) with known binding targets based on *cis*-regulatory motif analysis. The AUCell algorithm enabled quantification of each regulon. We used pyscenic (version 0.10.0) implemented via singularity v3.2 to perform single-cell regulatory network analysis and followed the published protocol steps.[83] We first run the python script 'arboreto_with_multiprocessing.py' using the 'grnboost2′ method followed by running 'pyscenic' using default parameters with the database file 'hg38__refseq-r80__10kb_up_and_down_tss.mc9nr.feather' and the motif information file 'motifs-v9-nr.hgnc-m0.001-o0.0.tbl'. Identified regulons from pyscenic were selected based on the average AUCell score across cells > 0.02 and the number of genes in each regulon > 10.

Quantitative real time PCR in shRNA experiments

In *DYRK1A* knockdown experiments, RNA was extracted using RNeasy micro kit using the protocol for low cell numbers (Qiagen #74004). RNA quality and quantification was performed on the nanodrop. Reverse transcription was performed with EvoScript Universal cDNA Master mix (Roche #07912455001) using 250-500 ng of RNA. qPCR was performed on a 7500 Real-Time PCR Machine using PCR Master

Mix (Applied Biosystems TaqMan Universal PCR Master Mix #4304437). DYRK1A (Hs00176369_m1 qPCR primer, ThermoFisher) expression levels were normalized to GAPDH (housekeeping gene, Hs00176369_m1 qPCR primer, ThermoFisher).

Dual luciferase transcriptional assay

In this STAT5 luciferase reporter assay system[84] in human embryonic kidney (HEK) 293T cells either WT *Jak2* or *Jak2V617F* are co-expressed. The thrombopoietin receptor (*TPOR*) is co-transfected in all conditions as HEK cells express low levels of endogenous *TPOR*. TPO is then added to the system to stimulate JAKSTAT signaling through binding to the TPOR. Transcriptional activation of STAT5 was analyzed via dual luciferase assay (Promega) perfomed in HEK293T cells by measuring the ratio of Spi-Luc reporter driven firefly and pRL-TK-driven renilla luciferase as previously reported.[84] HEK293T cells were transiently transfected using Lipofectamine 2000 reagent (ThermoFisher) with cDNAs coding for WT h-*TPOR*, murine (mu-) *Jak2* WT or *Jak2V617F*, mu-*Stat5b* (constitutively active or WT), *DYRK1A* WT or scramble control (**Supplementary Table 15**), or the empty pMX-IRES-GFP (PIG) vector, as indicated. Cells were stimulated or not with 10 ng/mL TPO (Miltenyi Biotec) for 24 hours. Luciferase activities were assayed 48 h posttransfection using the Spi-Luc reporter for STAT5 transcriptional activity.[85] pRL-TK was used as an internal transfection control.

Supplementary references

1. Harrison, C. J. *et al.* An international study of intrachromosomal amplification of chromosome 21 (iAMP21): Cytogenetic characterization and outcome. *Leukemia* **28**, 1015–1021 (2014).
2. Li, Y. *et al.* Constitutional and somatic rearrangement of chromosome 21 in acute lymphoblastic leukaemia. *Nature* **508**, 98–102 (2014).
3. Hormann, F. M. *et al.* Integrating copy number data of 64 iAMP21 BCP-ALL patients narrows the common region of amplification to 1.57 Mb. *Front Oncol* **13**, (2023).
4. Gao, Q. *et al.* The genomic landscape of acute lymphoblastic leukemia with intrachromosomal amplification of chromosome 21. *Blood Journal* (2023) doi:10.1182/blood.2022019094.
5. Xie, W. *et al.* iAMP21 in acute myeloid leukemia is associated with complex karyotype, TP53 mutation and dismal outcome. *Modern Pathology* **33**, 1389–1397 (2020).
6. Feki, A. & Hibaoui, Y. DYRK1A protein, a promising therapeutic target to improve cognitive deficits in down syndrome. *Brain Sciences* vol. 8 Preprint at https://doi.org/10.3390/brainsci8100187 (2018).
7. Martínez de Lagrán, M. *et al.* Motor phenotypic alterations in TgDyrk1a transgenic mice implicate DYRK1A in Down syndrome motor dysfunction. *Neurobiol Dis* **15**, 132–142 (2004).
8. Laham, A. J., Saber-Ayad, M. & El-Awady, R. DYRK1A: a down syndrome-related dual protein kinase with a versatile role in tumorigenesis. *Cellular and Molecular Life Sciences* vol. 78 603–619 Preprint at https://doi.org/10.1007/s00018-020-03626-4 (2021).
9. Malinge, S. *et al.* Increased dosage of the chromosome 21 ortholog Dyrk1a promotes megakaryoblastic leukemia in a murine model of down syndrome. *Journal of Clinical Investigation* **122**, 948–962 (2012).
10. Wegiel, J., Gong, C.-X. & Hwang, Y.-W. The role of DYRK1A in neurodegenerative diseases. *FEBS J* **278**, 236–245 (2011).
11. Kimura, R. *et al.* The DYRK1A gene, encoded in chromosome 21 Down syndrome critical region, bridges between β-amyloid production and tau phosphorylation in Alzheimer disease. *Hum Mol Genet* **16**, 15–23 (2007).
12. Pucelik, B., Barzowska, A., Dąbrowski, J. M. & Czarna, A. Diabetic kinome inhibitors—a new opportunity for β-cells restoration. *International Journal of Molecular Sciences* vol. 22 Preprint at https://doi.org/10.3390/ijms22169083 (2021).
13. De la Torre, R. *et al.* Epigallocatechin-3-gallate, a DYRK1A inhibitor, rescues cognitive deficits in Down syndrome mouse models and in humans. *Mol Nutr Food Res* **58**, 278–288 (2014).
14. Liu, T. *et al.* DYRK1A inhibitors for disease therapy: Current status and perspectives. *Eur J Med Chem* **229**, 114062 (2022).
15. Liu, Y. A. *et al.* Selective DYRK1A Inhibitor for the Treatment of Type 1 Diabetes: Discovery of 6-Azaindole Derivative GNF2133. *J Med Chem* **63**, 2958–2973 (2020).
16. Wang, E. *et al.* Modulation of RNA splicing enhances response to BCL2 inhibition in leukemia. *Cancer Cell* **41**, 164-180.e8 (2023).
17. Chen, C.-C., Amon, A., Hemann, M. & Rowe, R. G. Inherent Genome Instability Underlies Trisomy 21-Associated Myeloid Malignancies. *Blood* **142**, 1388–1388 (2023).

18. Pelleri, M. C. *et al.* Systematic reanalysis of partial trisomy 21 cases with or without Down syndrome suggests a small region on 21q22.13 as critical to the phenotype. *Hum Mol Genet* ddw116 (2016) doi:10.1093/hmg/ddw116.
19. Sebert, M. *et al.* Clonal hematopoiesis driven by chromosome 1q/MDM4 trisomy defines a canonical route toward leukemia in Fanconi anemia. *Cell Stem Cell* **30**, 153-170.e9 (2023).
20. Ceccaldi, R. *et al.* Bone marrow failure in Fanconi anemia is triggered by an exacerbated p53/p21 DNA damage response that impairs hematopoietic stem and progenitor cells. *Cell Stem Cell* **11**, 36–49 (2012).
21. Brady, S. W. *et al.* The genomic landscape of pediatric acute lymphoblastic leukemia. *Nat Genet* **54**, 1376–1389 (2022).
22. Xie, W. *et al.* iAMP21 in acute myeloid leukemia is associated with complex karyotype, TP53 mutation and dismal outcome. *Modern Pathology* **33**, 1389–1397 (2020).
23. Labuhn, M. *et al.* Mechanisms of Progression of Myeloid Preleukemia to Transformed Myeloid Leukemia in Children with Down Syndrome. *Cancer Cell* **36**, 123-138.e10 (2019).
24. Yi, Q. *et al.* Spectrum of BRAF Aberrations and Its Potential Clinical Implications: Insights From Integrative Pan-Cancer Analysis. *Front Bioeng Biotechnol* **10**, (2022).
25. Girish, V. *et al.* Oncogene-like addiction to aneuploidy in human cancers. *Science (1979)* (2023) doi:10.1126/science.adg4521.
26. Loh, P. R., Genovese, G. & McCarroll, S. A. Monogenic and polygenic inheritance become instruments for clonal selection. *Nature* **584**, 136–141 (2020).
27. Loh, P. R. *et al.* Insights into clonal haematopoiesis from 8,342 mosaic chromosomal alterations. *Nature* **559**, 350–355 (2018).
28. Venkatraman, E. S. & Olshen, A. B. A faster circular binary segmentation algorithm for the analysis of array CGH data. *Bioinformatics* **23**, 657–663 (2007).
29. Olshen, A. B., Venkatraman, E. S., Lucito, R. & Wigler, M. Circular binary segmentation for the analysis of array-based DNA copy number data. *Biostatistics* **5**, 557–572 (2004).
30. Lawrence, M. *et al.* Software for Computing and Annotating Genomic Ranges. *PLoS Comput Biol* **9**, (2013).
31. Bashton, M. *et al.* Concordance of copy number abnormality detection using SNP arrays and Multiplex Ligation-dependent Probe Amplification (MLPA) in acute lymphoblastic leukaemia. *Sci Rep* **10**, (2020).
32. Mermel, C. H. *et al.* GISTIC2.0 facilitates sensitive and confident localization of the targets of focal somatic copy-number alteration in human cancers. *Genome Biol* **12**, (2011).
33. Rausch, T. *et al.* Genome Sequencing of Pediatric Medulloblastoma Links Catastrophic DNA Rearrangements with TP53 Mutations. *Cell* **148**, 59–71 (2012).
34. Korbel, J. O. & Campbell, P. J. Criteria for inference of chromothripsis in cancer genomes. *Cell* vol. 152 1226–1236 Preprint at https://doi.org/10.1016/j.cell.2013.02.023 (2013).
35. Tyner, J. W. *et al.* Functional genomic landscape of acute myeloid leukaemia. *Nature* **562**, 526–531 (2018).
36. Ley, T. J. Genomic and Epigenomic Landscapes of Adult De Novo Acute Myeloid Leukemia. *New England Journal of Medicine* **368**, 2059–2074 (2013).
37. Tazi, Y. *et al.* Unified classification and risk-stratification in Acute Myeloid Leukemia. *Nat Commun* **13**, 4622 (2022).
38. Heath, A. P. *et al.* The NCI Genomic Data Commons. *Nat Genet* **53**, 257–262 (2021).

39. Cerami, E. *et al.* The cBio Cancer Genomics Portal: An Open Platform for Exploring Multidimensional Cancer Genomics Data. *Cancer Discov* **2**, 401–404 (2012).
40. Medina-Martínez, J. S. *et al.* Isabl Platform, a digital biobank for processing multimodal patient data. *BMC Bioinformatics* **21**, (2020).
41. Li, H. & Durbin, R. Fast and accurate short read alignment with Burrows–Wheeler transform. *Bioinformatics* **25**, 1754–1760 (2009).
42. Pedersen, B. S. & Quinlan, A. R. Mosdepth: quick coverage calculation for genomes and exomes. *Bioinformatics* **34**, 867–868 (2018).
43. Nik-Zainal, S. *et al.* The life history of 21 breast cancers. *Cell* **149**, 994–1007 (2012).
44. Kim, S. *et al.* Strelka2: fast and accurate calling of germline and somatic variants. *Nat Methods* **15**, 591–594 (2018).
45. Jones, D. *et al.* cgpCaVEManWrapper: Simple Execution of CaVEMan in Order to Detect Somatic Single Nucleotide Variants in NGS Data. *Curr Protoc Bioinformatics* **56**, (2016).
46. van der Auwera, G. & O'Connor, B. D. *Genomics in the Cloud: Using Docker, GATK, and WDL in Terra*. (O'Reilly Media, Incorporated, 2020).
47. Ye, K., Schulz, M. H., Long, Q., Apweiler, R. & Ning, Z. Pindel: a pattern growth approach to detect break points of large deletions and medium sized insertions from paired-end short reads. *Bioinformatics* **25**, 2865–2871 (2009).
48. Wala, J. A. *et al.* SvABA: genome-wide detection of structural variants and indels by local assembly. *Genome Res* **28**, 581–591 (2018).
49. Cameron, D. L. *et al.* GRIDSS: sensitive and specific genomic rearrangement detection using positional de Bruijn graph assembly. *Genome Res* **27**, 2050–2060 (2017).
50. McLaren, W. *et al.* The Ensembl Variant Effect Predictor. *Genome Biol* **17**, 122 (2016).
51. Menzies, A. *et al.* VAGrENT: Variation Annotation Generator. *Curr Protoc Bioinformatics* **52**, (2015).
52. Dentro, S. & Wedge, D. dpclust3p: DPClust pre-processing. R package version 1.0.8. (2020).
53. Dentro, S. C., Wedge, D. C. & Van Loo, P. Principles of Reconstructing the Subclonal Architecture of Cancers. *Cold Spring Harb Perspect Med* **7**, a026625 (2017).
54. Jakobsdottir, G. M., Dentro, S. C., Bristow, R. G. & Wedge, D. C. AmplificationTimeR: An R Package for Timing Sequential Amplification Events. *Bioinformatics* (2024) doi:10.1093/bioinformatics/btae281.
55. Cortés-Ciriano, I. *et al.* Comprehensive analysis of chromothripsis in 2,658 human cancers using whole-genome sequencing. *Nat Genet* **52**, 331–341 (2020).
56. Kidd, J. M. *et al.* A Human Genome Structural Variation Sequencing Resource Reveals Insights into Mutational Mechanisms. *Cell* **143**, 837–847 (2010).
57. De Coster, W., D'Hert, S., Schultz, D. T., Cruts, M. & Van Broeckhoven, C. NanoPack: visualizing and processing long-read sequencing data. *Bioinformatics* **34**, 2666–2669 (2018).
58. Sedlazeck, F. J. *et al.* Accurate detection of complex structural variations using single-molecule sequencing. *Nat Methods* **15**, 461–468 (2018).
59. Giurgiu, M. *et al.* Reconstructing extrachromosomal DNA structural heterogeneity from long-read sequencing data using Decoil. *Genome Res* gr.279123.124 (2024) doi:10.1101/gr.279123.124.
60. inferCNV of the Trinity CTAT Project. https://github.com/broadinstitute/inferCNV.
61. Gao, T. *et al.* Haplotype-aware analysis of somatic copy number variations from single-cell transcriptomes. *Nat Biotechnol* (2022) doi:10.1038/s41587-022-01468-y.

62. Rodriguez-Meira, A. *et al.* Single-cell multi-omics identifies chronic inflammation as a driver of TP53-mutant leukemic evolution. *Nat Genet* **55**, 1531–1541 (2023).
63. Giustacchini, A. *et al.* Single-cell transcriptomics uncovers distinct molecular signatures of stem cells in chronic myeloid leukemia. *Nat Med* **23**, 692–702 (2017).
64. Rodriguez-Meira, A., O'Sullivan, J., Rahman, H. & Mead, A. J. TARGET-Seq: A Protocol for High-Sensitivity Single-Cell Mutational Analysis and Parallel RNA Sequencing. *STAR Protoc* **1**, 100125 (2020).
65. Rodriguez-Meira, A. *et al.* Unravelling Intratumoral Heterogeneity through High-Sensitivity Single-Cell Mutational Analysis and Parallel RNA Sequencing. *Mol Cell* **73**, 1292-1305.e8 (2019).
66. Dobin, A. *et al.* STAR: ultrafast universal RNA-seq aligner. *Bioinformatics* **29**, 15–21 (2013).
67. Liao, Y., Smyth, G. K. & Shi, W. featureCounts: an efficient general purpose program for assigning sequence reads to genomic features. *Bioinformatics* **30**, 923–930 (2014).
68. Love, M. I., Huber, W. & Anders, S. Moderated estimation of fold change and dispersion for RNA-seq data with DESeq2. *Genome Biol* **15**, 550 (2014).
69. Subramanian, A. *et al.* Gene set enrichment analysis: A knowledge-based approach for interpreting genome-wide expression profiles. *Proceedings of the National Academy of Sciences* **102**, 15545–15550 (2005).
70. Mootha, V. K. *et al.* PGC-1α-responsive genes involved in oxidative phosphorylation are coordinately downregulated in human diabetes. *Nat Genet* **34**, 267–273 (2003).
71. Langmead, B. Aligning Short Sequencing Reads with Bowtie. *Curr Protoc Bioinformatics* **32**, (2010).
72. Li, H. *et al.* The Sequence Alignment/Map format and SAMtools. *Bioinformatics* **25**, 2078–2079 (2009).
73. Kent, W. J. *et al.* The Human Genome Browser at UCSC. *Genome Res* **12**, 996–1006 (2002).
74. Zhang, Y. *et al.* Model-based Analysis of ChIP-Seq (MACS). *Genome Biol* **9**, R137 (2008).
75. Corces, M. R. *et al.* The chromatin accessibility landscape of primary human cancers. *Science (1979)* **362**, (2018).
76. Heinz, S. *et al.* Simple Combinations of Lineage-Determining Transcription Factors Prime cis-Regulatory Elements Required for Macrophage and B Cell Identities. *Mol Cell* **38**, 576–589 (2010).
77. Iness, A. N. *et al.* The cell cycle regulatory DREAM complex is disrupted by high expression of oncogenic B-Myb. *Oncogene* **38**, 1080–1092 (2019).
78. Butler, A., Hoffman, P., Smibert, P., Papalexi, E. & Satija, R. Integrating single-cell transcriptomic data across different conditions, technologies, and species. *Nat Biotechnol* **36**, 411–420 (2018).
79. Granja, J. M. *et al.* Single-cell multiomic analysis identifies regulatory programs in mixed-phenotype acute leukemia. *Nat Biotechnol* **37**, 1458–1465 (2019).
80. Hao, Y. *et al.* Integrated analysis of multimodal single-cell data. *Cell* **184**, 3573-3587.e29 (2021).
81. Oetjen, K. A. *et al.* Human bone marrow assessment by single-cell RNA sequencing, mass cytometry, and flow cytometry. *JCI Insight* **3**, (2018).
82. van Galen, P. *et al.* Single-Cell RNA-Seq Reveals AML Hierarchies Relevant to Disease Progression and Immunity. *Cell* **176**, 1265-1281.e24 (2019).
83. Van de Sande, B. *et al.* A scalable SCENIC workflow for single-cell gene regulatory network analysis. *Nat Protoc* **15**, 2247–2276 (2020).

84. Girardot, M. *et al.* Persistent STAT5 activation in myeloid neoplasms recruits p53 into gene regulation. *Oncogene* **34**, 1323–1332 (2015).
85. Wood, T. J. J. *et al.* Specificity of transcription enhancement via the STAT responsive element in the serine protease inhibitor 2.1 promoter. *Mol Cell Endocrinol* **130**, 69–81 (1997).
