# Supplementary material for: Chromothripsis-associated chromosome 21 amplification orchestrates transformation to blast-phase MPN through targetable overexpression of DYRK1A
Source: Nat Genet. 2025 Jun 9;57(6):1478–92. doi: 10.1038/s41588-025-02190-6 (PMC12165854; doi:10.1038/s41588-025-02190-6)
Supplement: Supplementary file 2 — Reporting Summary [file 41588_2025_2190_MOESM2_ESM.pdf]

nature portfolio

Corresponding author(s): Charlotte K Brierley, John Crispino, Adam J Mead

Last updated by author(s): Mar 27, 2025

Reporting Summary

Nature Portfolio wishes to improve the reproducibility of the work that we publish. This form provides structure for consistency and transparency in reporting. For further information on Nature Portfolio policies, see our Editorial Policies and the Editorial Policy Checklist.

Statistics

For all statistical analyses, confirm that the following items are present in the figure legend, table legend, main text, or Methods section.

n/a	Confirmed	
☐	☒	The exact sample size (*n*) for each experimental group/condition, given as a discrete number and unit of measurement
☐	☒	A statement on whether measurements were taken from distinct samples or whether the same sample was measured repeatedly
☐	☒	The statistical test(s) used AND whether they are one- or two-sided<br>*Only common tests should be described solely by name; describe more complex techniques in the Methods section.*
☐	☒	A description of all covariates tested
☐	☒	A description of any assumptions or corrections, such as tests of normality and adjustment for multiple comparisons
☐	☒	A full description of the statistical parameters including central tendency (e.g. means) or other basic estimates (e.g. regression coefficient) AND variation (e.g. standard deviation) or associated estimates of uncertainty (e.g. confidence intervals)
☐	☒	For null hypothesis testing, the test statistic (e.g. *F*, *t*, *r*) with confidence intervals, effect sizes, degrees of freedom and *P* value noted<br>*Give P values as exact values whenever suitable.*
☒	☐	For Bayesian analysis, information on the choice of priors and Markov chain Monte Carlo settings
☒	☐	For hierarchical and complex designs, identification of the appropriate level for tests and full reporting of outcomes
☐	☒	Estimates of effect sizes (e.g. Cohen's *d*, Pearson's *r*), indicating how they were calculated

Our web collection on statistics for biologists contains articles on many of the points above.

Software and code

Policy information about availability of computer code

Data collection

- Targeted myeloid-panel sequencing libraries were generated from bulk genomic DNA using a TruSeq Custom Amplicon panel (Illumina) and were sequenced on a MiSeq (Illumina) instrument.

- SNP-Array data was generated through hybridization of bulk genomic DNA to an Illumina Infinium OmniExpress v1.3 BeadChips Array and SNP-CGH CytoScan HD Array

- Whole genome sequencing was performed on bulk genomic DNA isolated from CD3+ depleted cells. Samples underwent PCR-free library preparation prior to 80-100X whole genome sequencing on an Illumina NovaSeq S4

-- Mini-bulk RNA sequencing was performed on 200 CD34+ lineage negative cells isolated by FACS, with cDNA libraries generated using the Smart-Seq 2 kit prior to pooling followed by sequencing on the NextSeq 500 platform.

-Mini-bulk ATAC-sequencing was performed on 1000 CD34+ lineage negative cells isolated by FACS followed by the Tn5 transposase reaction. cDNA libraries were generated, pooled and sequenced on the NextSeq 500 platform.

-10X single-cell RNA-seq was performed on CD34+ lineage negative cells. Samples were processed according to the 10x protocol using the Chromium Single Cell 30 library and Gel Bead Kits v3.0.

- Flow cytometry data was collected using BD FACS Diva Software (v8.0.2).

- Data collection methods are fully described in the manuscript

Data analysis

- Targeted myeloid-panel sequencing data: SOPHiA DDM® (Sophia Genetics) and an in-house software GRIO-Dx®.
- SNP Array data : Mocha WDL pipeline v2021-01-20 (https://software.broadinstitute.org/software/mocha/mocha.20210120.wdl), GISTIC2,

March 2021

Chromosome Analysis Suite software package (v4.1, Affymetrix), SHAPEIT v4.1.3.
-WGS: Isabl platform pipeline and interface (https://www.isabl.io/). BWA-mem (v0.7.17) as a part of the pcap-core v2.18.2 wrapper (https://github.com/cancerit/PCAP-core). Mosdepth 4 (https://github.com/brentp/mosdepth). cgpBattenberg (v1.4.0, https://github.com/cancerit/cgpBattenberg). Strelka2 (v2.9.1 with manta v1.3.1, https://github.com/Illumina/strelka), MuTect2 (gatk:v4.0.1.2, https://github.com/broadinstitute/gatk), CaVEMan (cgpCavemanWrapper v1.7.5, https://github.com/cancerit/cgpCaVEManWrapper), cgpCavemanPostprocessing (v1.5.2, https://github.com/cancerit/cgpCaVEManPostProcessing). Pindel (cgpPindel v1.5.4, https://github.com/cancerit/cgpPindel), SvABA (~v1.0.0 commit 47c7a88, https://github.com/walaj/svaba), GRIDSS (v2.2.2, https://github.com/PapenfussLab/gridss), BRASS (v4.0.5 with GRASS v1.1.6, https://github.com/cancerit/BRASS), ClusterSV (v1.0.0, https://github.com/cancerit/ClusterSV/).
- Amplification timing analysis: dpclust3p R package (v1.0.8, https://github.com/Wedge-lab/dpclust3p) and AmplificationTimeR R package (v1.1.1, https://github.com/Wedge-lab/AmplificationTimeR).
- Single cell TARGETSeq data: SingCellaR v1.2.0 was used for data analysis and plotting (https://supatt-lab.github.io/SingCellaR.Doc/index.html). CNA inference was performed using numbat (v1.4.0).
- RNA/ATAC-seq analysis: bcl2fastq v2.20.0.422, FastQC v0.11.5 (https://github.com/s-andrews/FastQC), TrimGalore v0.6.5 (https://github.com/FelixKrueger/TrimGalore), STAR v2.6.1d, Subread (v2.0.0), Bowtie2 v2.4.2, MarkDuplicates module from Picard v2.3.2, Samtools v1.9, ATACseqQC R package (v1.14.4) MACS2 v2.2.7.1, ChIPseeker R package (v1.34.1), TxDb.Hsapiens.UCSC.hg.knownGene (v3.2.2). GenomicRanges (v1.50.2) R package. DESeq2 R package (v1.28.1). Homer (v20201202). FactorMineR (v2.8), factoextra (v1.0.7), ComplexHeatmap R package (v 2.14.0), EnhancedVolcano R package (v1.16.0), pheatmap v1.0.12, GSEA software (Broad Institute; v4.3.2, RRID: SCR_003199).
- 10x Genomics single-cell RNA-seq analysis: bcl2fastq (2.20.0.422) and Cell Ranger software (version 7.0.0) from 10x Genomics. Cite-seq-count/1.4.4 Souporcell pipeline v2.0, troublet v2.4. Seurat v.4.0.1 in R 4.0.4, numbat v1.3.0, pyscenic (v0.10.0) implemented via singularity v3.2
- Synergy analysis: SynergyFinder web application v2 (https://github.com/IanevskiAleksandr/SynergyFinder).
-Genotyping of CRISPR clones: CRIS.py
-Western blot imaging: Odyssey CLx Imaging System (LI-COR).
- Flow cytometry data was analyzed using FlowJo (version 10.7.1.1, BD Biosciences) software.
-`Statistical analyses: R v4.0.4, Prism v7 or later, SPSS v29.0.0.
- Custom codes available at https://github.com/wimm-hscb-lab-published/ upon publication

For manuscripts utilizing custom algorithms or software that are central to the research but not yet described in published literature, software must be made available to editors and reviewers. We strongly encourage code deposition in a community repository (e.g. GitHub). See the Nature Portfolio guidelines for submitting code & software for further information.

Data

Policy information about availability of data

All manuscripts must include a data availability statement. This statement should provide the following information, where applicable:

- Accession codes, unique identifiers, or web links for publicly available datasets
- A description of any restrictions on data availability
- For clinical datasets or third party data, please ensure that the statement adheres to our policy

All raw and processed sequencing data generated in this study will be made publically available at the NCBI Gene Expression Omnibus (GEO; https://www.ncbi.nlm.nih.gov/geo/) under accession number GSE228060 for CRISPR KO clones, GSE240407 for RNA/ATAC and GSE292030 for single cell primary patient data. The TARGET-seq single cell dataset is available in raw and processed format at GEO accession number GSE226340 and SRA accession number PRJNA930152. The raw and processed SNP array data, and single cell (10X) Seurat object generated in this manuscript is available at Zenodo at the following DOI: 10.5281/zenodo.14749740.

Whole genome sequencing data has been deposited at the European Genome-phenome Archive (EGA), which is hosted by the EBI and the CRG, under accession number EGAS00001007483.
Other publically available datasets accessed:

Tazi et al (Nature Communications, 2022)
BeatAML (Tyner et al, Nature, 2018)
The Cancer Genome Atlas (TCGA) (Ley et al, N Engl J Med, 2013)
The DepMap Cancer Dependency Map (https://depmap.org/portal/)
The Cancer Cell Line Encyclopedia (https://sites.broadinstitute.org/ccle/)
Custom scripts will be available at https://github.com/wimm-hscb-lab-published/Brierley_NG_chr21amp

Field-specific reporting

Please select the one below that is the best fit for your research. If you are not sure, read the appropriate sections before making your selection.

☒ Life sciences ☐ Behavioural & social sciences ☐ Ecological, evolutionary & environmental sciences

For a reference copy of the document with all sections, see nature.com/documents/nr-reporting-summary-flat.pdf

Life sciences study design

All studies must disclose on these points even when the disclosure is negative.

Sample size	Sample size was determined based on similar studies in the field and availability of samples.

Data exclusions	Data which didn't reach quality control parameters (as detailed in Methods section) were excluded from the analysis.
Replication	In vitro and in vivo experiments in the manuscript were repeated to reach 3 biological replicates in at least 2 independent experiments. Attempts at replication were successful. Details on numbers of replicates are provided in the relevant legend and/or methods section.
Randomization	Patients samples were separated according to their diagnosis and chr21amp status,randomization was not appropriate. Mice were allocated randomly to control or KO groups.
Blinding	Blinding was not relevant for single cell data, as the information on chr21amp status was required for analysis. For mouse experiments, blinding was performed for analysis of FACS data with an anonymized identification number for each mouse.

Reporting for specific materials, systems and methods

We require information from authors about some types of materials, experimental systems and methods used in many studies. Here, indicate whether each material, system or method listed is relevant to your study. If you are not sure if a list item applies to your research, read the appropriate section before selecting a response.

Materials & experimental systems

n/a	Involved in the study	
☐	☒	Antibodies
☐	☒	Eukaryotic cell lines
☒	☐	Palaeontology and archaeology
☐	☒	Animals and other organisms
☐	☒	Human research participants
☒	☐	Clinical data
☒	☐	Dual use research of concern

Methods

n/a	Involved in the study	
☒	☐	ChIP-seq
☐	☒	Flow cytometry
☒	☐	MRI-based neuroimaging

Antibodies

Antibodies used	All antibodies used for the study are detailed in Extended Data Table 12 and 16.<br><br>Antibodies used:<br>CD34-APC efluor780, eBiosciences (Thermo Fisher Scientific), Cat# 47-0349-42 RRID AB_2573956<br>Lineage antibody cocktail (CD3, CD14, CD16, CD19, CD20, CD56)-BV510, Biolegend, Cat# 328122 RRID AB_2561420<br>7AAD, BD Pharminogen, Cat# 51-68981E<br>γ-H2AX Alexa488, Abcam, Cat# ab195188<br>Propidium iodide, eBioscience, Cat# 88-8007-72<br>Annexin V, eBioscience, Cat# 88-8007-72<br>Total-Seq A Hashtag 1, Biolegend, Cat# 394601<br>Total-Seq A Hashtag 2, Biolegend, Cat# 394603<br>Total-Seq A Hashtag 4, Biolegend, Cat# 394607<br>Total-Seq A Hashtag3, Biolegend, Cat# 394605<br>DYRK1A, Abnova, Cat# H00001859-M01<br>HSC70 (HRP/AF680/AF790), Santa Cruz Biotechnology, Cat# sc-7298<br>IRDye® 680RD secondary, LI-COR Biosciences, Cat# 926-68073 & Cat# 926-68072<br>IRDye® 800CW secondary, LI-COR Biosciences, Cat# 926-32213 & Cat# 926-32212<br>beta-Actin, Santa Cruz Biotechnology, Cat# sc-47778 AF680<br>STAT3, Cell Signaling Technology, Cat# 9139S<br>pSTAT3-Y705, Cell Signaling Technology, Cat# 9145S<br>Alexa Fluor® 488 Anti-gamma H2A.X (phospho S139), Adcam, Cat# ab195188<br>LIN52, Invitrogen, Cat# PA5-64882<br>Phospho-S28-LIN52 , Gift from L. Litovchick (www.nature.com/articles/s41388-018-0490-y)<br>FOXO1, Cell Signaling technology , Cat# 2880S<br>GRB2, BD Biosciences, Cat# 610111
Validation	Human and mouse antibodies were already validated, titrated and referenced in peer-reviewed publications, as described on the suppliers' websites (Biolegend, eBiosciences, BD horizon, BD Biosciences, BD Pharmingen, Beckman Coulter). Combination of antibodies for human hematopoietic stem cells and progenitors have been already tested in previous publications (Psaila et al, Mol cel 2020, Rodriguez-Meira et al, Nature Genetics 2023).

Eukaryotic cell lines

Policy information about cell lines

Cell line source(s)	American Type Culture Collection (ATCC) for SET2, HEL and HEK293T cell lines

Authentication	STR testing was performed and confirmed expected identity.
Mycoplasma contamination	Cell lines underwent regular mycoplasma testing, which were negative.
Commonly misidentified lines (See ICLAC register)	None used.

Animals and other organisms

Policy information about studies involving animals; ARRIVE guidelines recommended for reporting animal research

Laboratory animals	Mouse housing was carried out in individually ventilated cages (19-24°C, humidity 40-65%, 12/12 light dark cycle). Enrichment was done with nesting and bedding material. Mice were fed on standard croquettes, and supplemented with nutritionally complete gel diet after irradiation and in case of weight loss. Mice were maintained on a specific and opportunistic pathogen free health status. NOD SCID Gamma (NSG) mice obtained from The Jackson Laboratory and maintained in St Jude Animal Resource Center, St Jude Children's Hospital, Memphis, TN, USA. Female mice aged 5 weeks.
Wild animals	This study did not involve wild animals.
Field-collected samples	Study did not involve field-collected samples.
Ethics oversight	The animal study was approved by the St. Jude Children's Research Hospital Institutional Animal Care and Use Committee. All experiments were performed at St Jude under animal protocol number 657-100655.

Note that full information on the approval of the study protocol must also be provided in the manuscript.

Human research participants

Policy information about studies involving human research participants

Population characteristics	Patients samples were selected based on their pathology (patients with blast phase myelopoliferative neoplasm). Healthy donor control samples were also used for the study. The gender was not take into account to select the population of interest. Data on individual samples is provide in Extended Data Table 1.
Recruitment	Samples were collected as part of patients' routine clinical care through previously established research study approvals as detailed below.
Ethics oversight	Peripheral blood and bone marrow samples were collected from BP-MPN patients and healthy donors from the PHAZAR study (Approval REC: 4/WM/1260; IRAS: 163072, 19 Jan 2015), the INForMeD Study (REC: 199833, 26 July 2016, University of Oxford), and the INSERM biobank (approved by the Inserm Institutional Review Board Ethical Committee, project C19-73, agreement 21-794, CODECOH n°DC-2020-4324). Patients and normal donors provided written informed consent in accordance with the Declaration of Helsinki for sample collection and use in research. The current study does not report outcomes of a clinical trial.

Note that full information on the approval of the study protocol must also be provided in the manuscript.

Flow Cytometry

Plots

Confirm that:

☒ The axis labels state the marker and fluorochrome used (e.g. CD4-FITC).

☒ The axis scales are clearly visible. Include numbers along axes only for bottom left plot of group (a 'group' is an analysis of identical markers).

☒ All plots are contour plots with outliers or pseudocolor plots.

☒ A numerical value for number of cells or percentage (with statistics) is provided.

Methodology

Sample preparation	Human or cell line samples were stained in PBS + 5% FCS (respectively) with several antibodies, incubated during 20min at RT and washed before being analyzed.<br><br>All methods for sample preparation are fully described in the methods section of the manuscript.
Instrument	Cells were analyzed on a BD Fortessa X20 (BD Biosciences) or Attune NxT (Invitrogen, Model AFC2) instrument.<br>Cells were sorted on a BD Fusion I or Fusion II instruments (Becton Dickinson).
Software	Analysis of the flow cytometry data was performed using FlowJo (version 10.7.1, BD Biosciences) softwares.
Cell population abundance	Human and mouse haematopoietic stem and progenitor (HSPC) populations represent minor cell types (in the majority of cases, less than 1-5% of the total sample), except when they display a competitive advantage in the context of leukemic

	transformation. Sorting was performed in purity mode for bulk experiments Post-sort purify was checked by sorting 100 cells from selected HSPC fractions (e.g. Lin-CD34+ cells for human experiments) into an eppendorf tube containing 100 uL sorting buffer and analyzing the number of cells included within the same immunophenotype. Post-sort purity was consistently above 95%.
Gating strategy	For HSPC analyses, viable single cells were gated on expression of CD34+Lin- expression using a well-established lineage panel (Psaila et al, Mol Cell, 2020, Rodriguez-Meira et al, Mol Cell, 2019, Rodriguez-Meira et al, Nat Genetics, 2023) For viability analyses, single cells were gated in quadrants by Annexin-V/Propidium Iodide expression as per manufacturer's instruction (https://www.thermofisher.com/order/catalog/product/88-8007-74). Gating strategies are outlined in Extended Data Fig6B,8A &B

☒ Tick this box to confirm that a figure exemplifying the gating strategy is provided in the Supplementary Information.
