# Supplementary material for: Chromothripsis-associated chromosome 21 amplification orchestrates transformation to blast-phase MPN through targetable overexpression of DYRK1A
Source: Nat Genet. 2025 Jun 9;57(6):1478–92. doi: 10.1038/s41588-025-02190-6 (PMC12165854; doi:10.1038/s41588-025-02190-6)

Fig. 5A

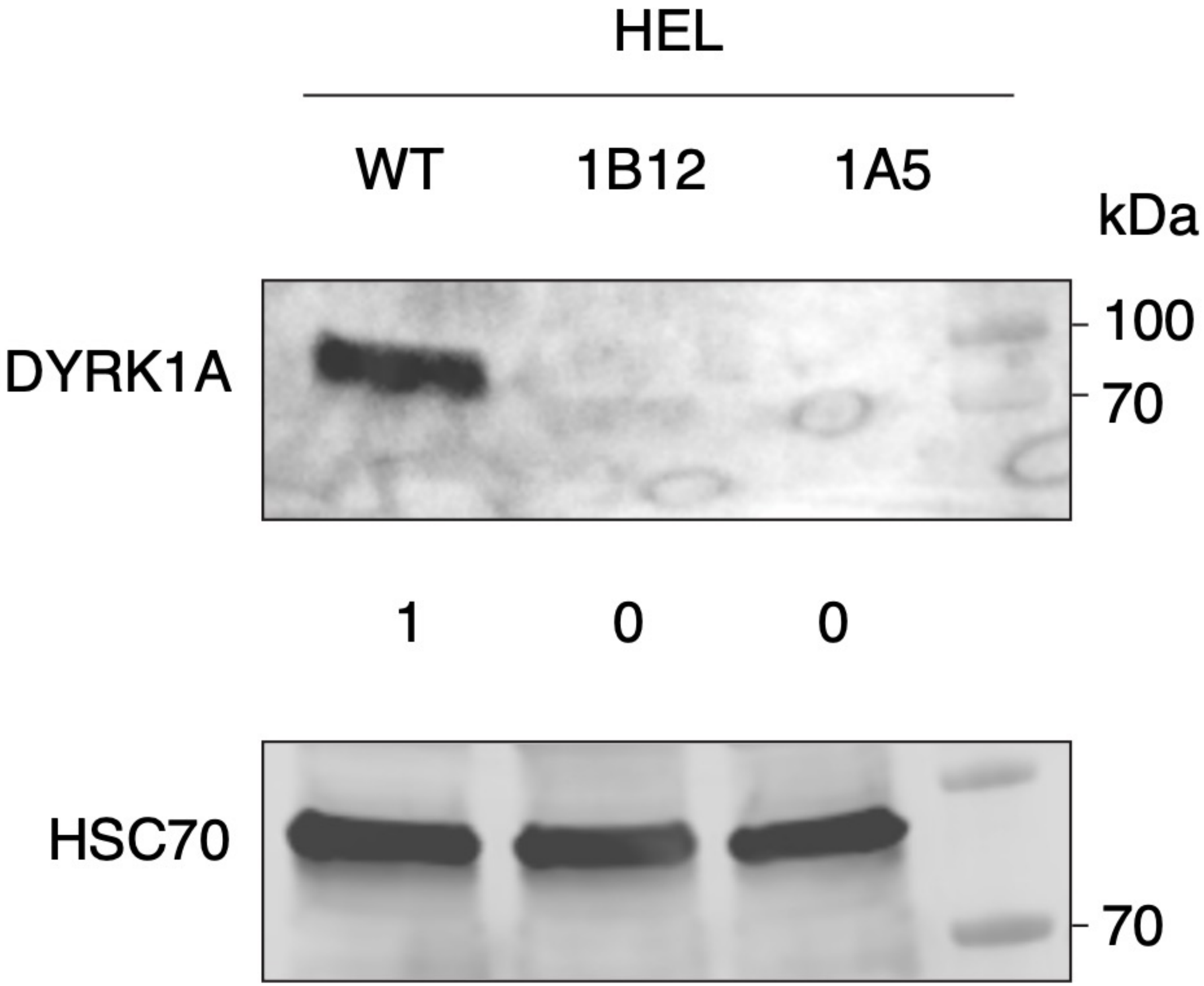

Extended Data Fig 5B

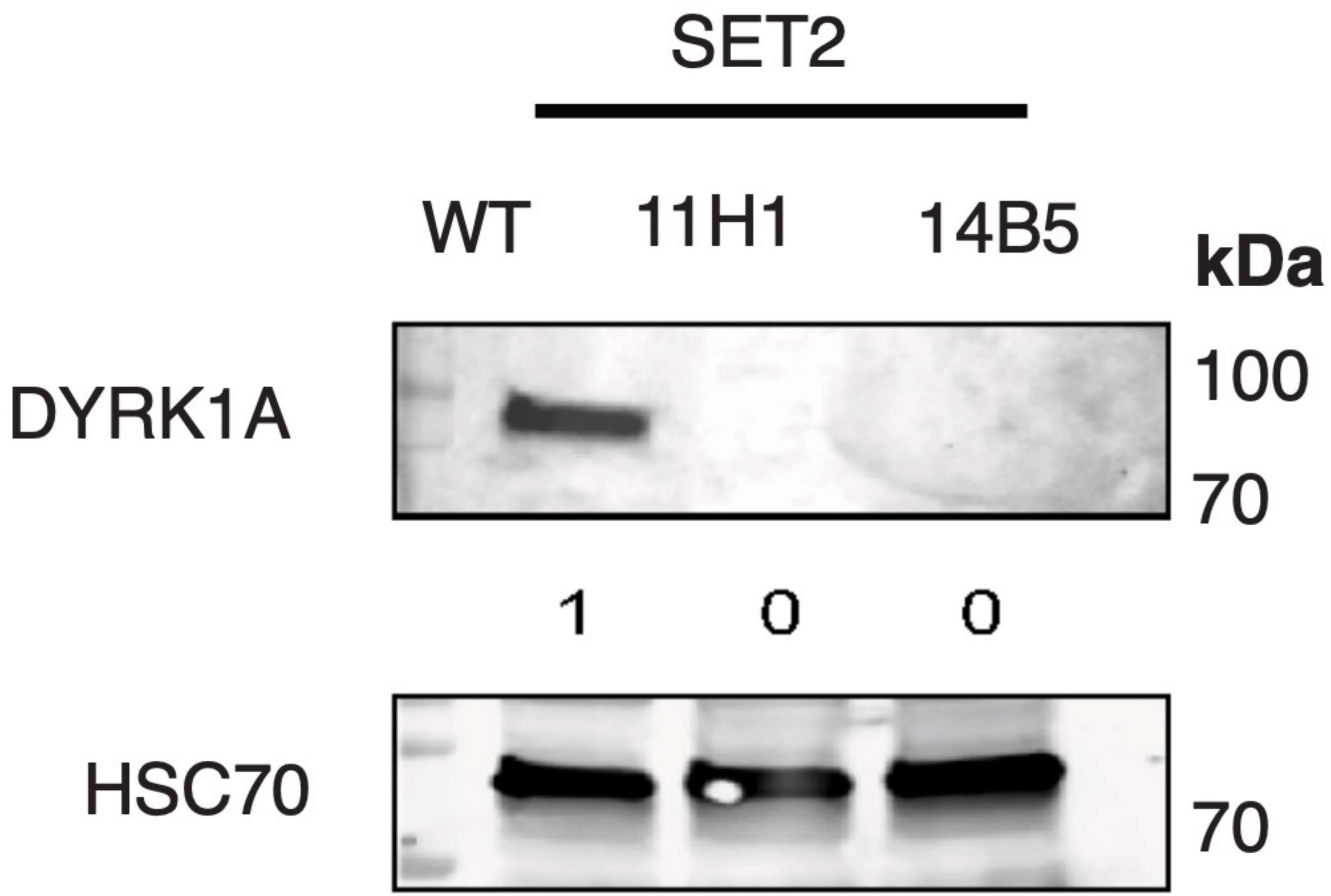

Whole membrane for Fig. 5A and Extended Data Fig 5B

Probed for DYRK1A

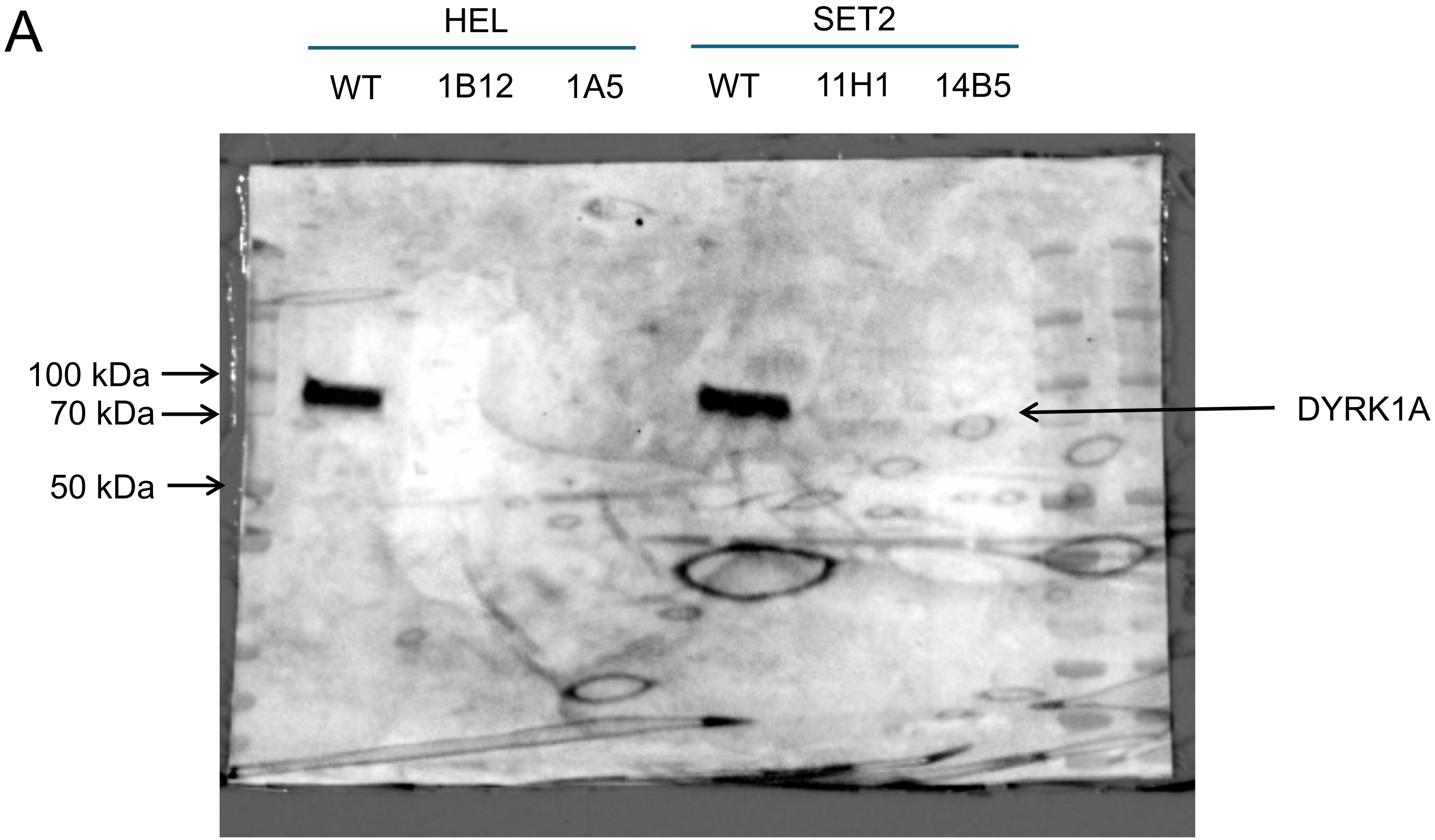

Whole membrane for Fig. 5A and Extended Data Fig 5B

Probed for HSC70
Alexa 790

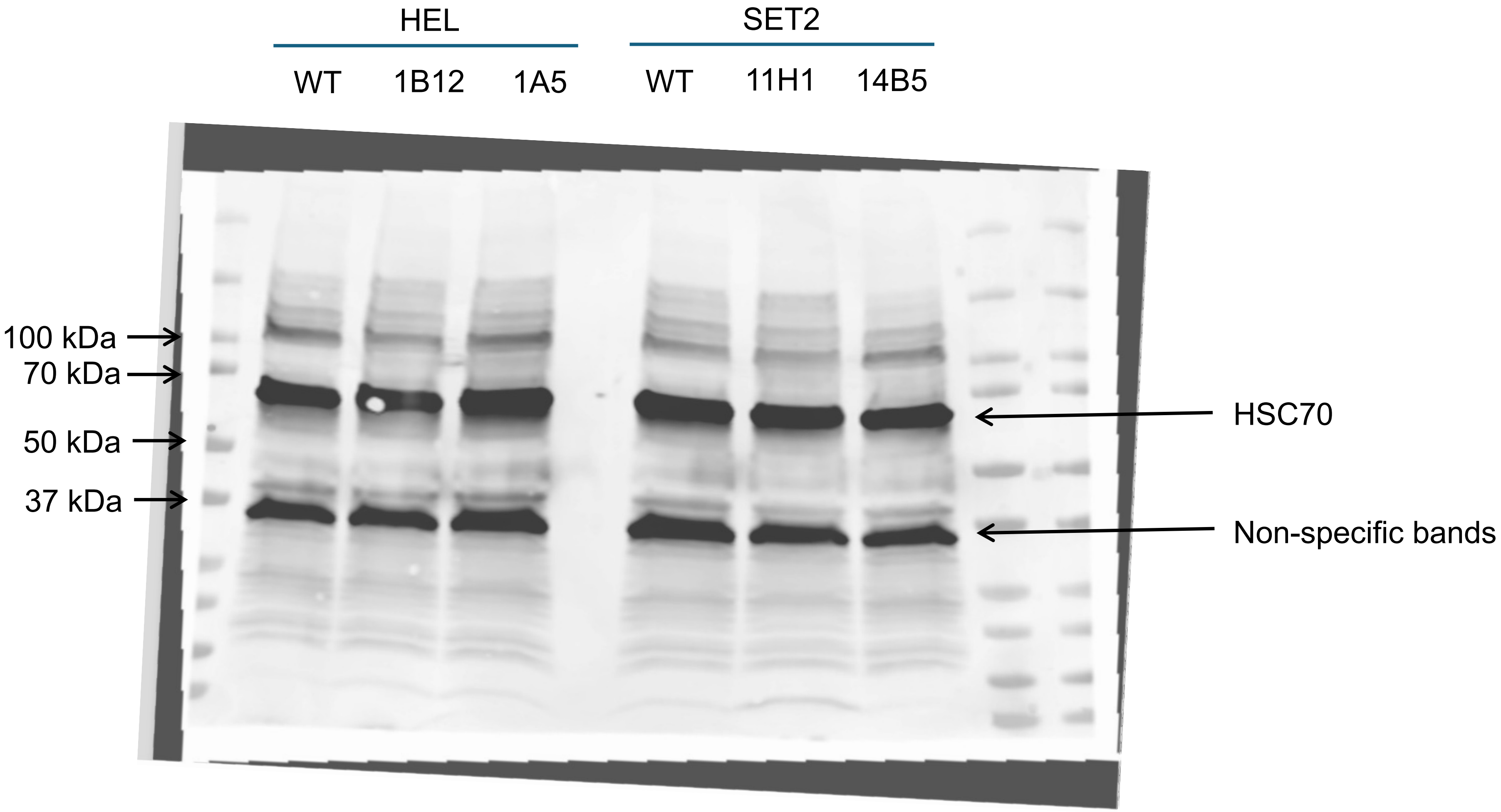

Fig. 5C

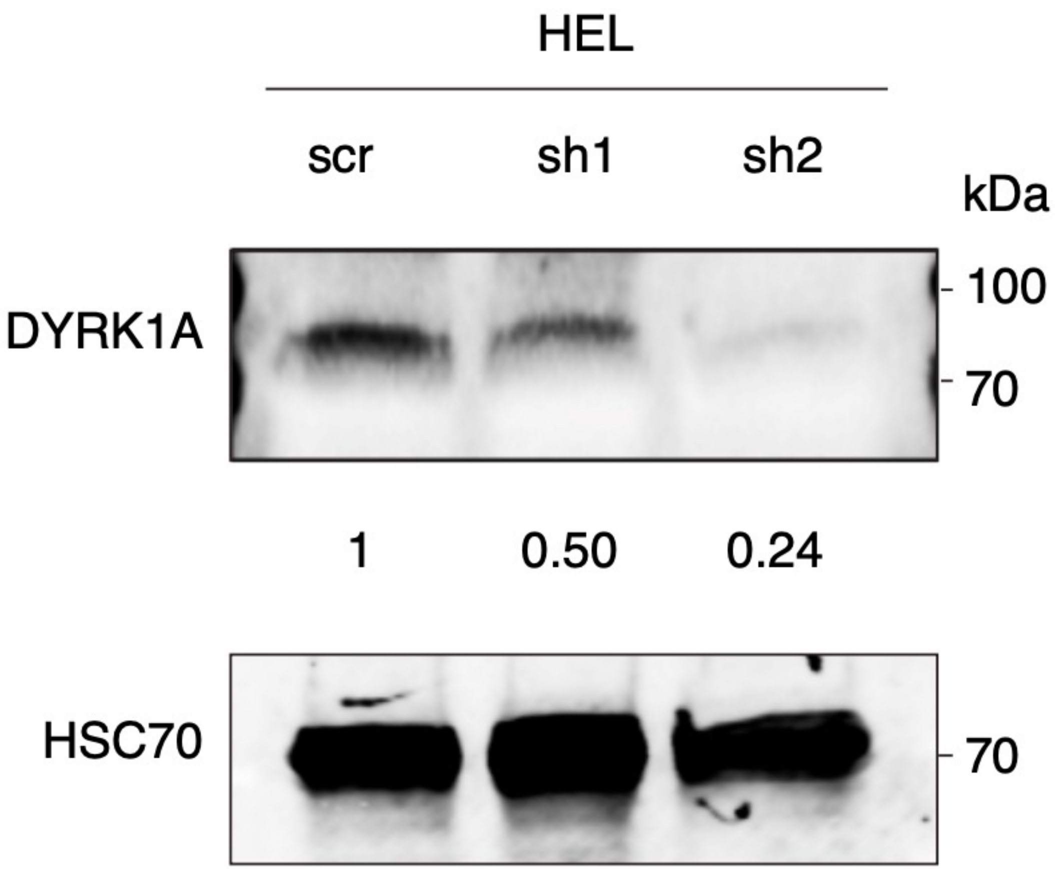

Whole membrane for
Fig. 5C

Probed for DYRK1A

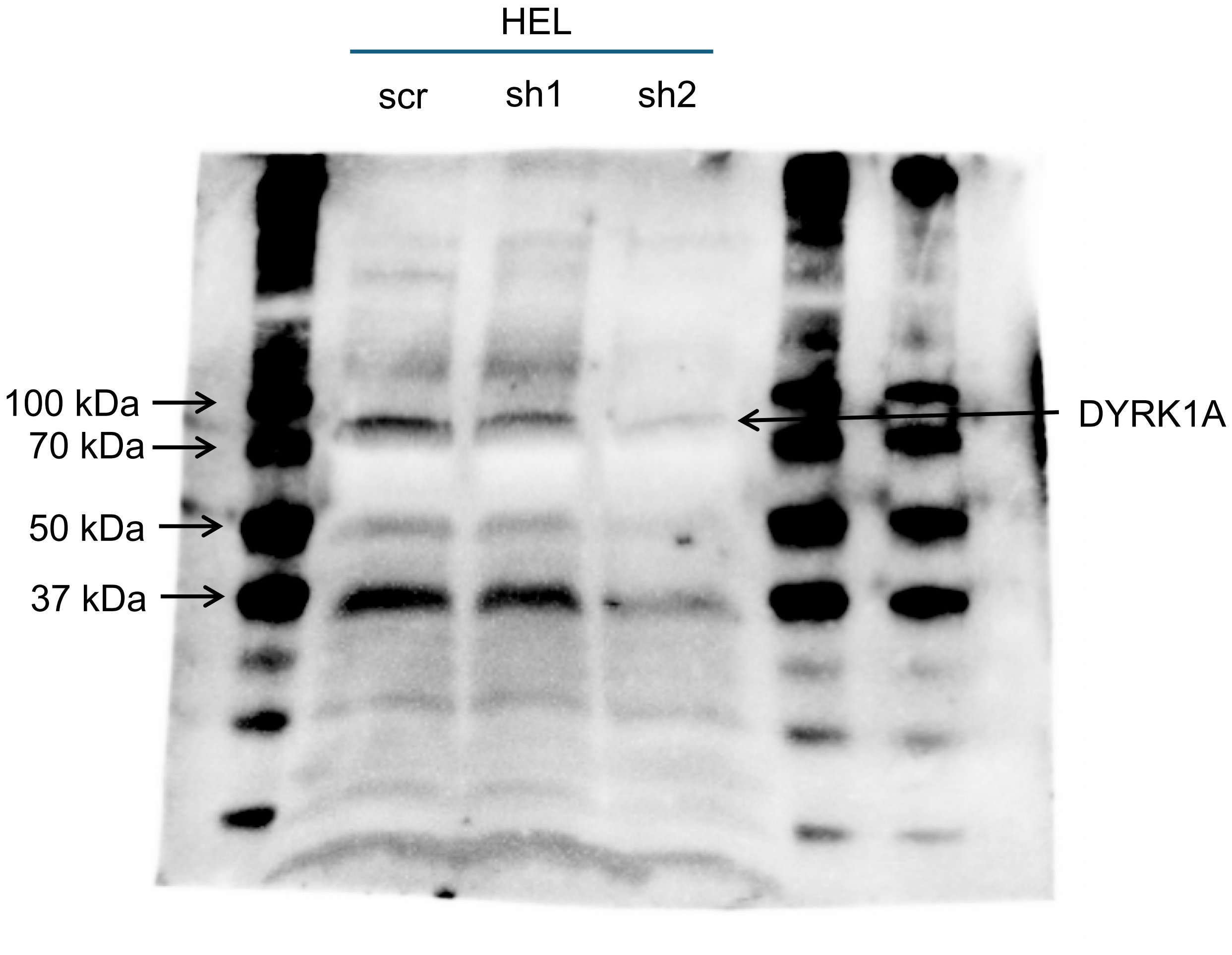

Whole membrane for
Fig. 5C

Probed for HSC70 Alexa
790

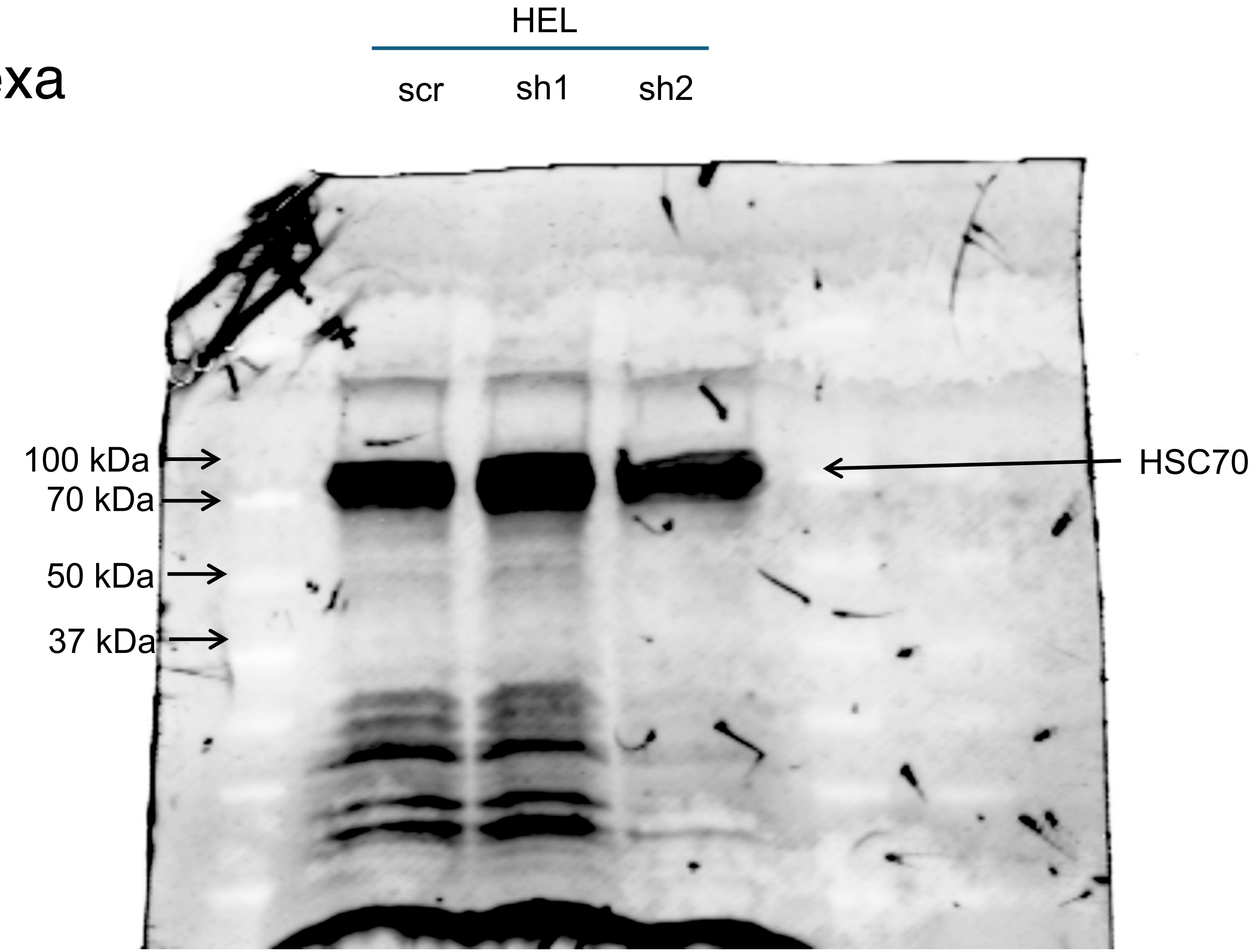

Extended Data Fig 5D

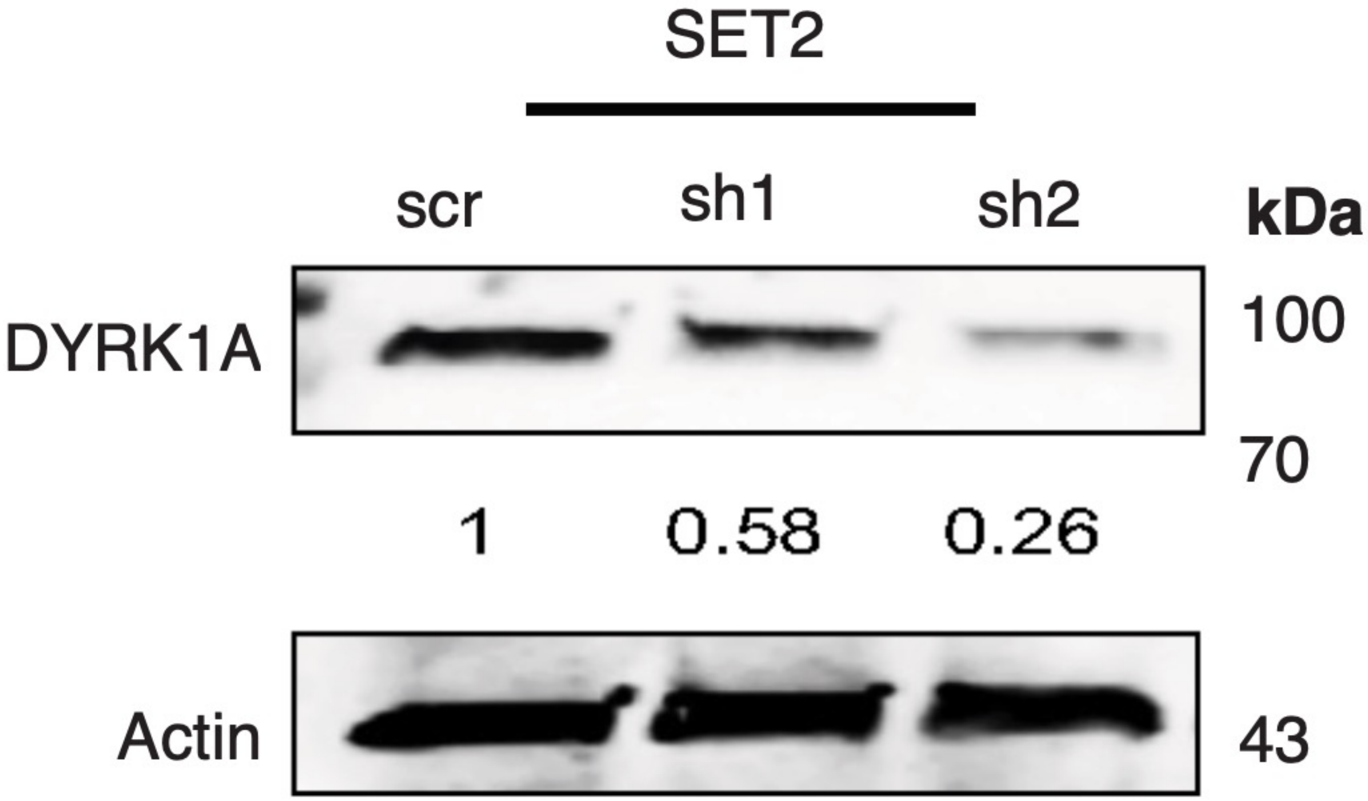

Whole membrane for
Extended Data Fig 5D

Probed for DYRK1A

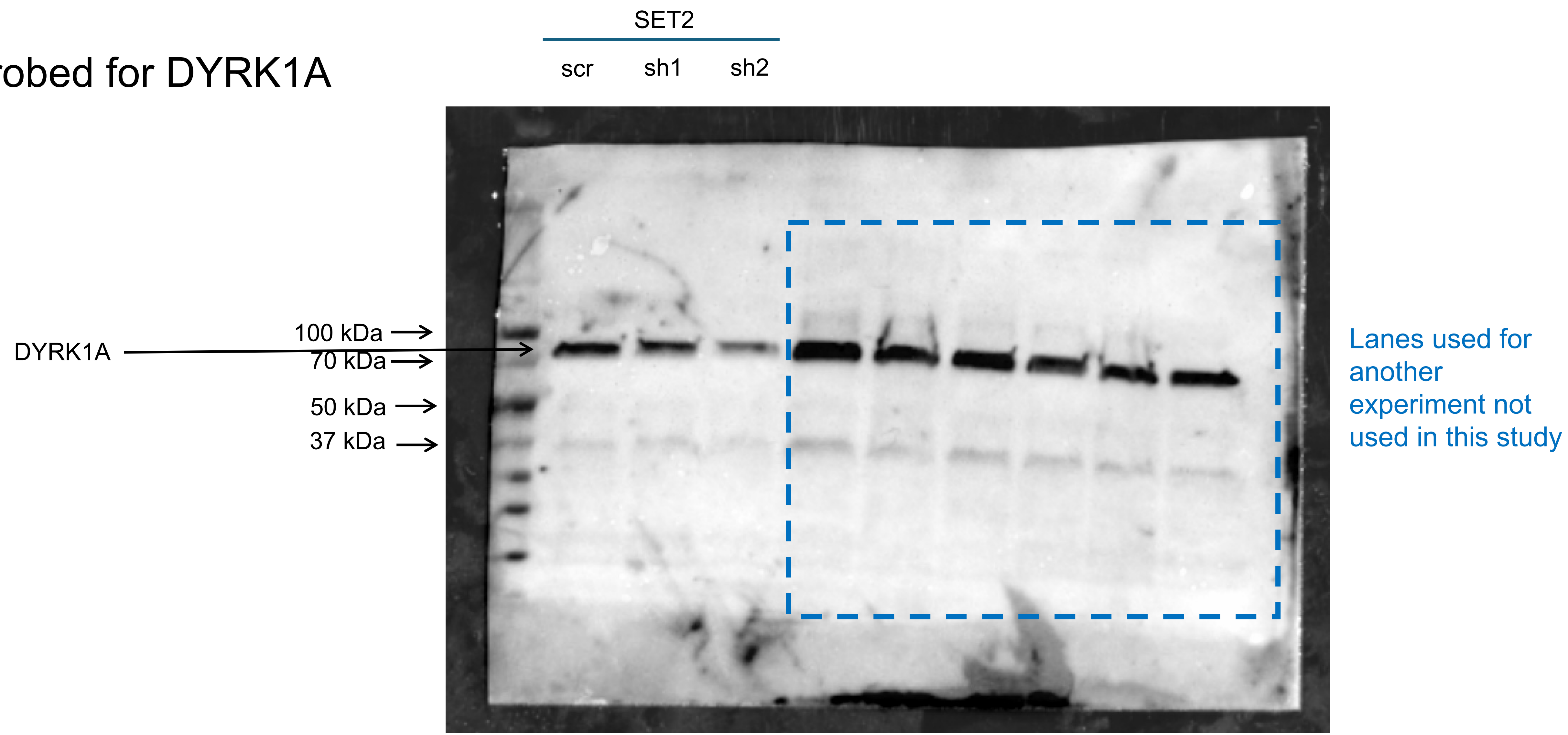

Whole membrane for Extended Data Fig 5D

Probed for Beta-actin Alexa 790

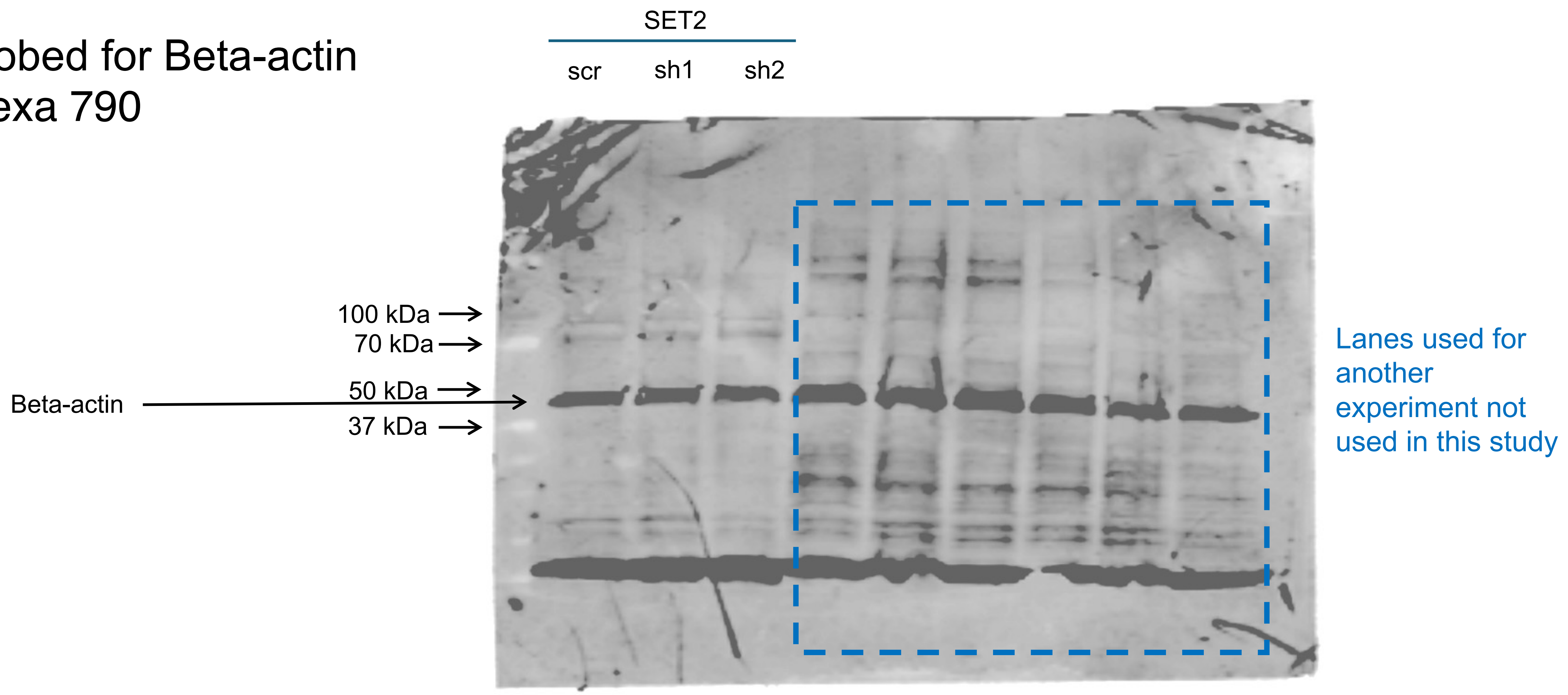

Extended Data Fig 5G

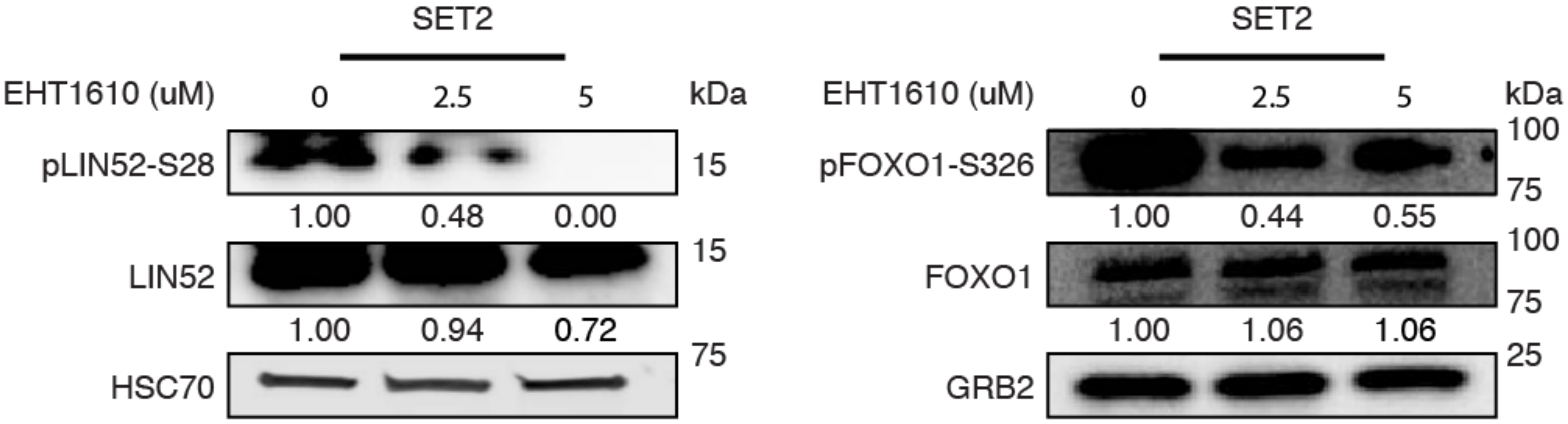

Extended Data Fig 5G

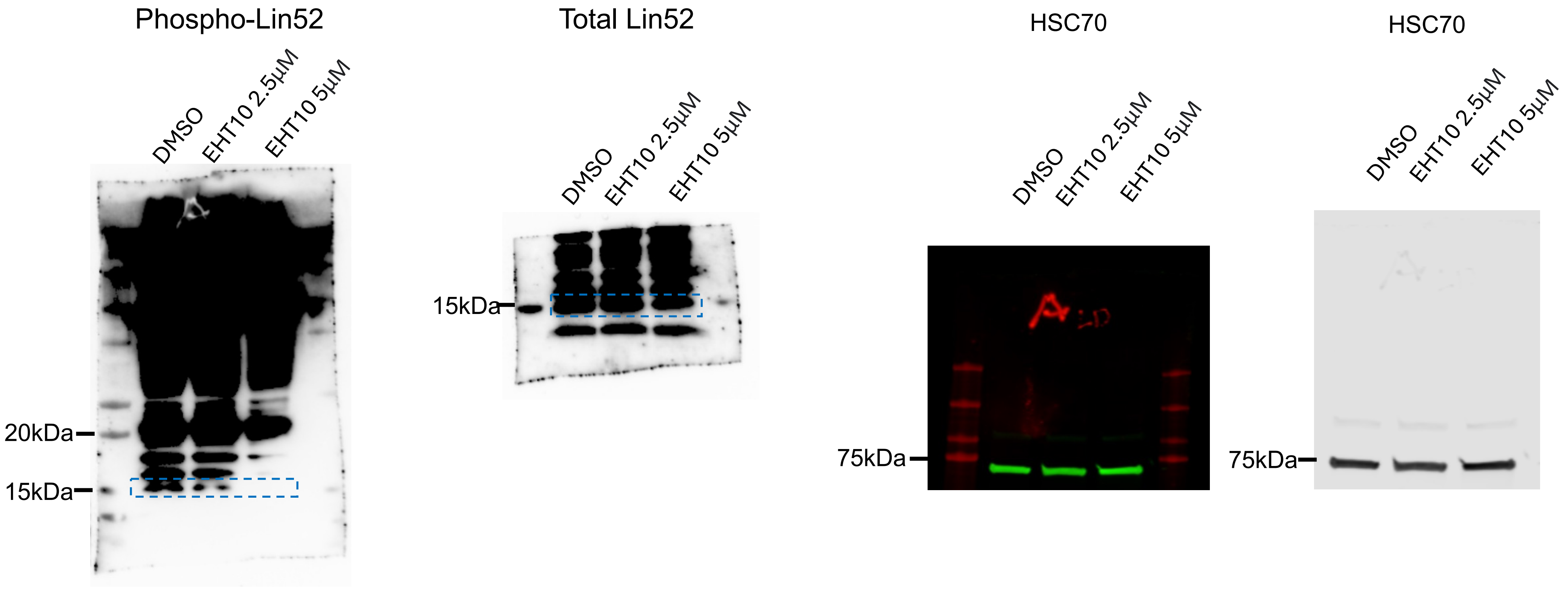

Extended Data Fig 5G

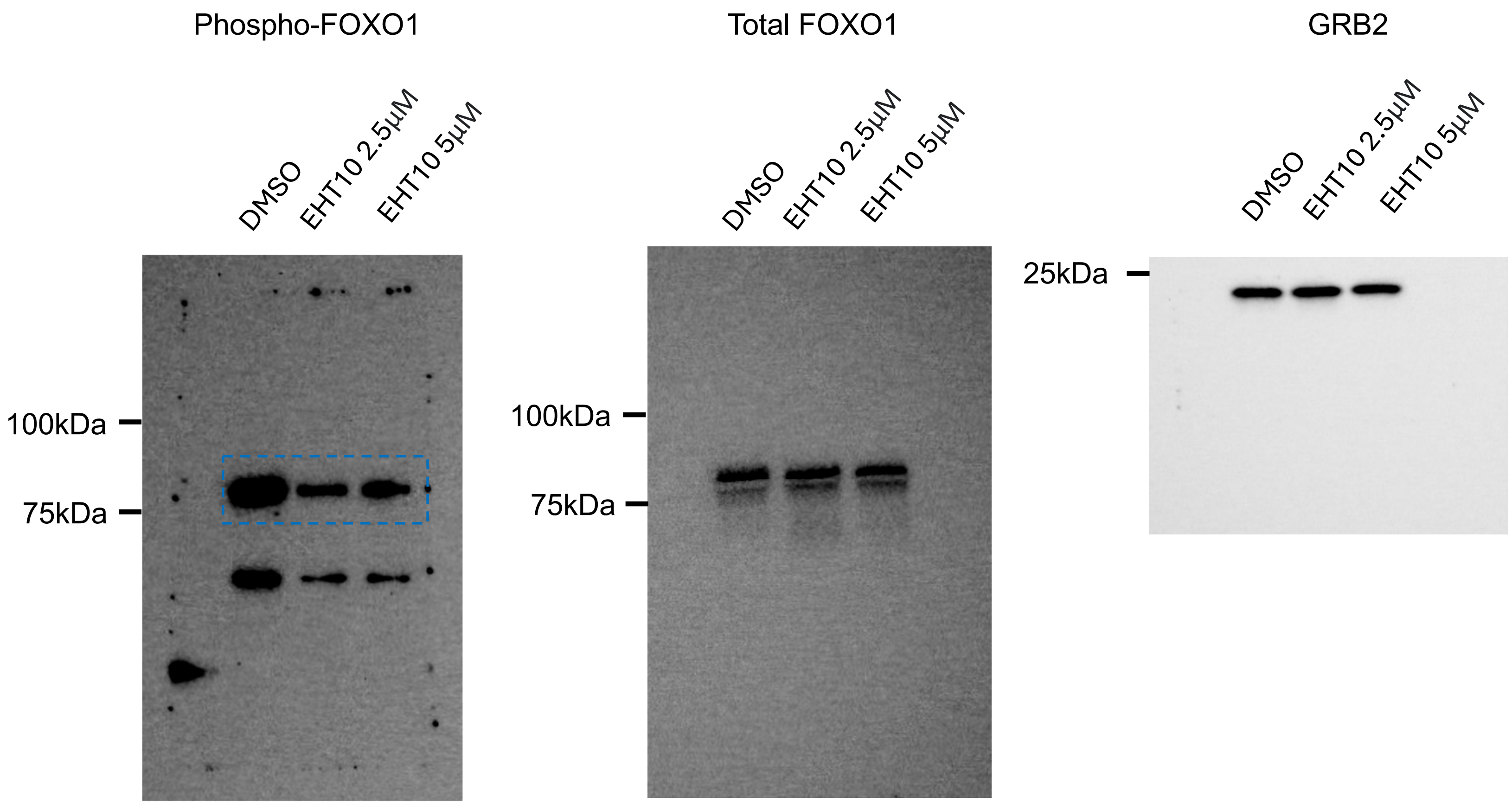

Extended Data Fig 7B

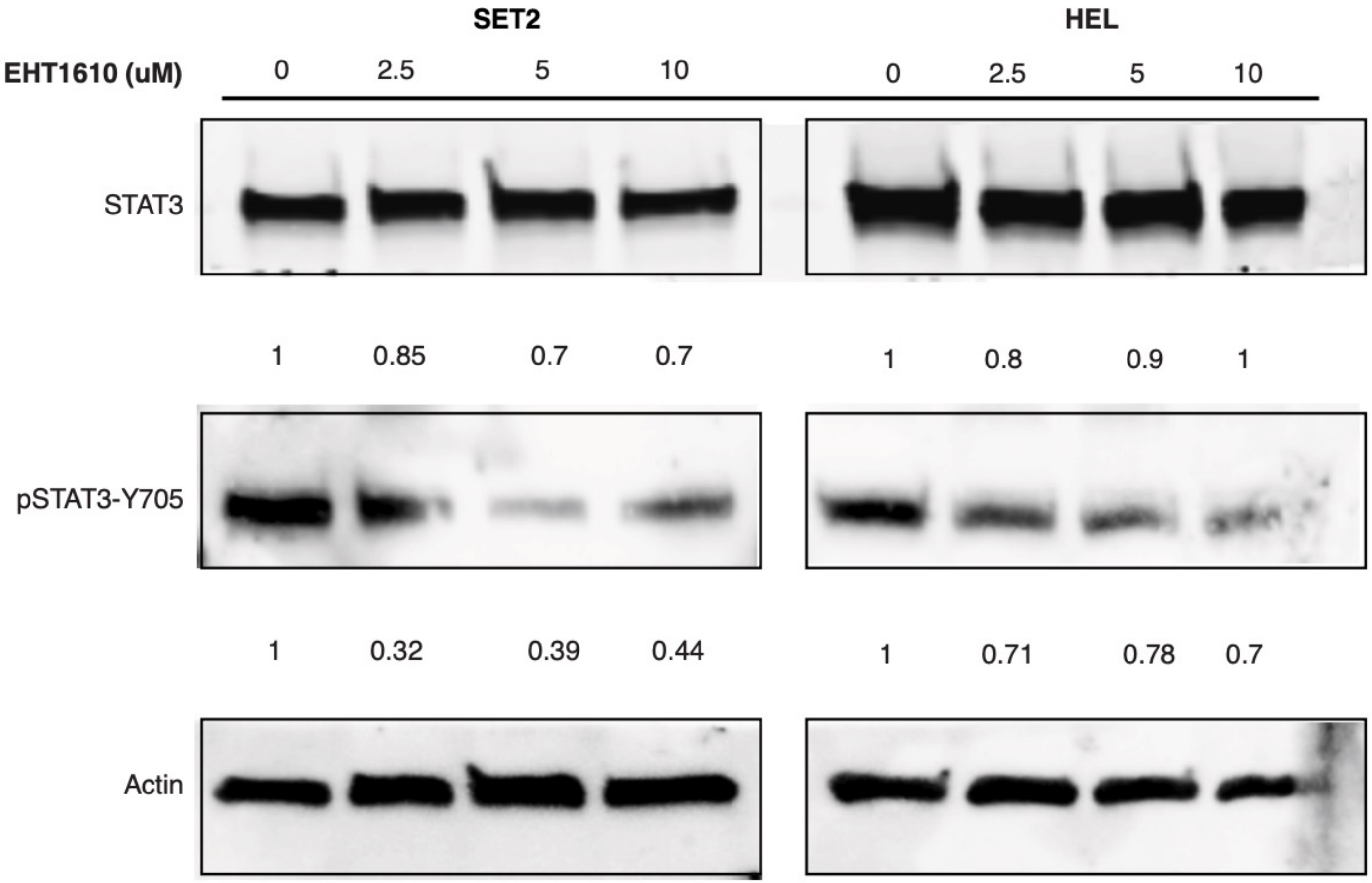

Extended Data Fig 7B

Upper part of the same membrane was probed for total STAT3

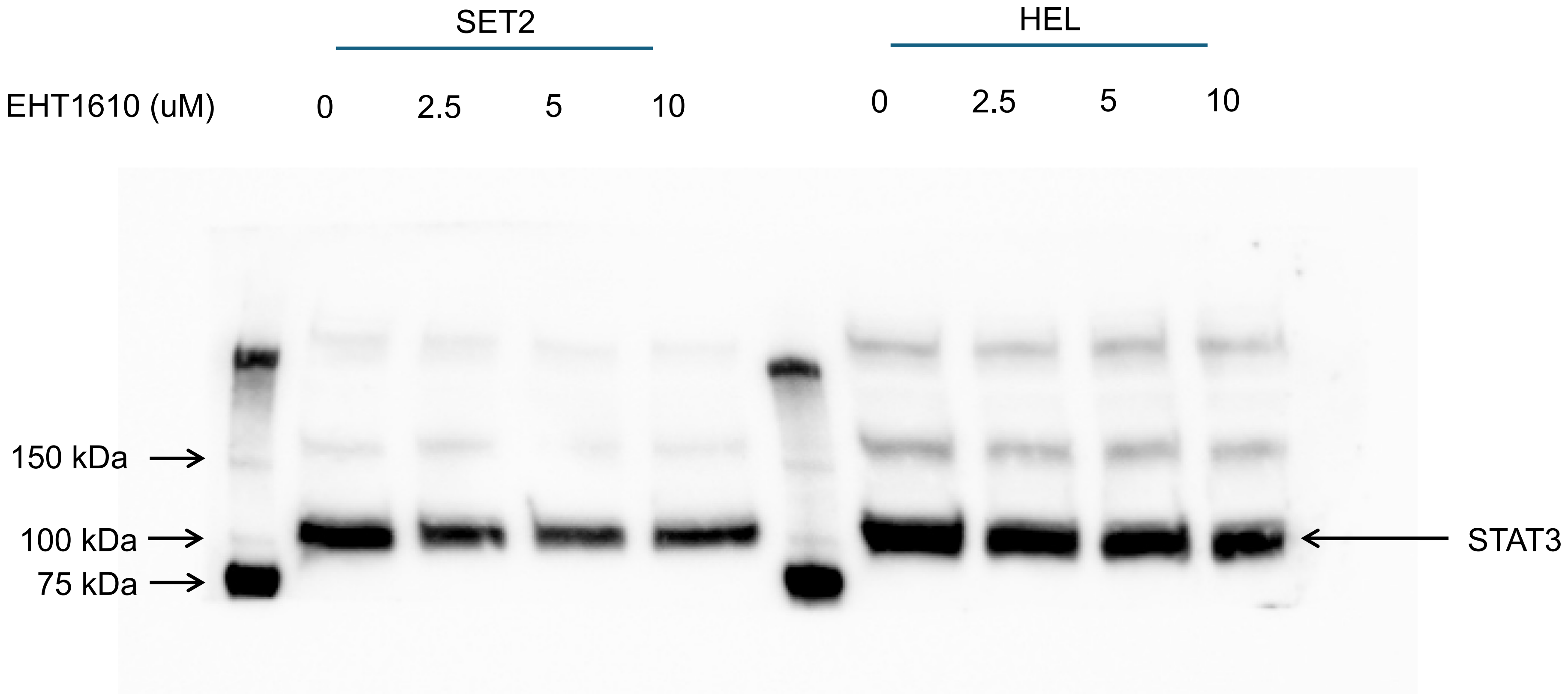

Extended Data Fig 7B

Upper part of the same membrane was stripped and then probed for pSTAT3-Y705

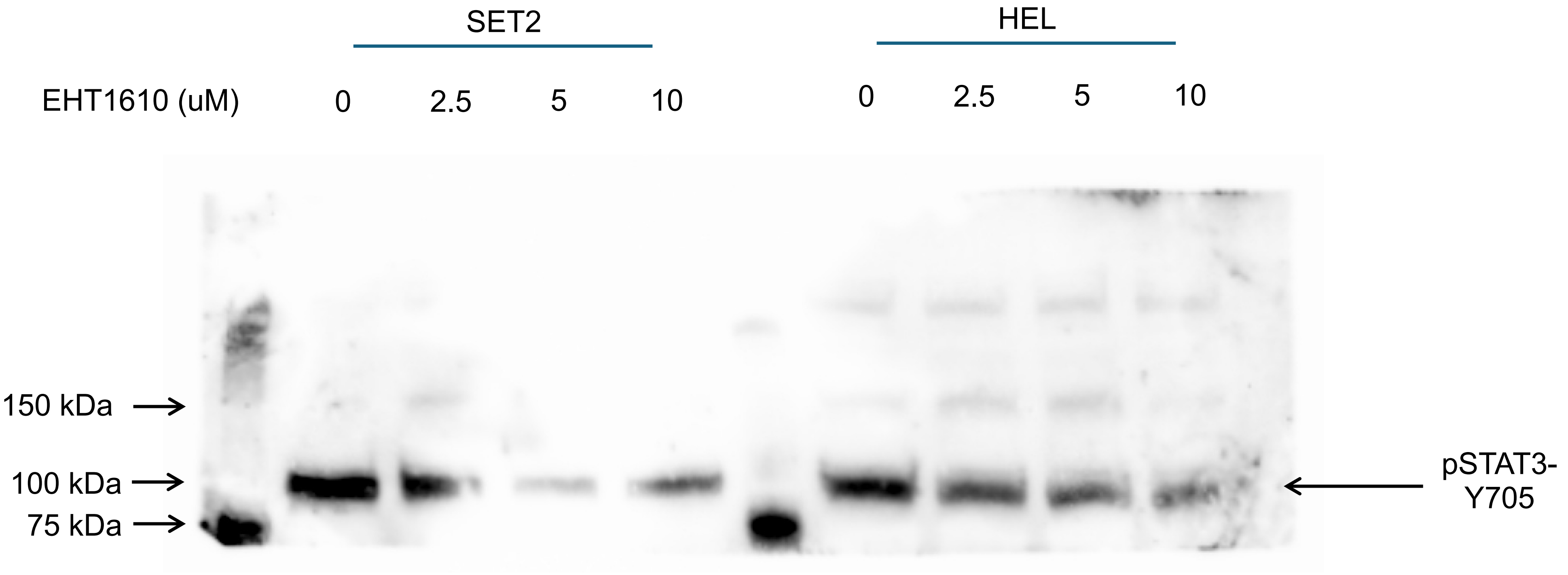

Extended Data Fig 7B

Lower part of the same membrane was probed for pSTAT3-Y705

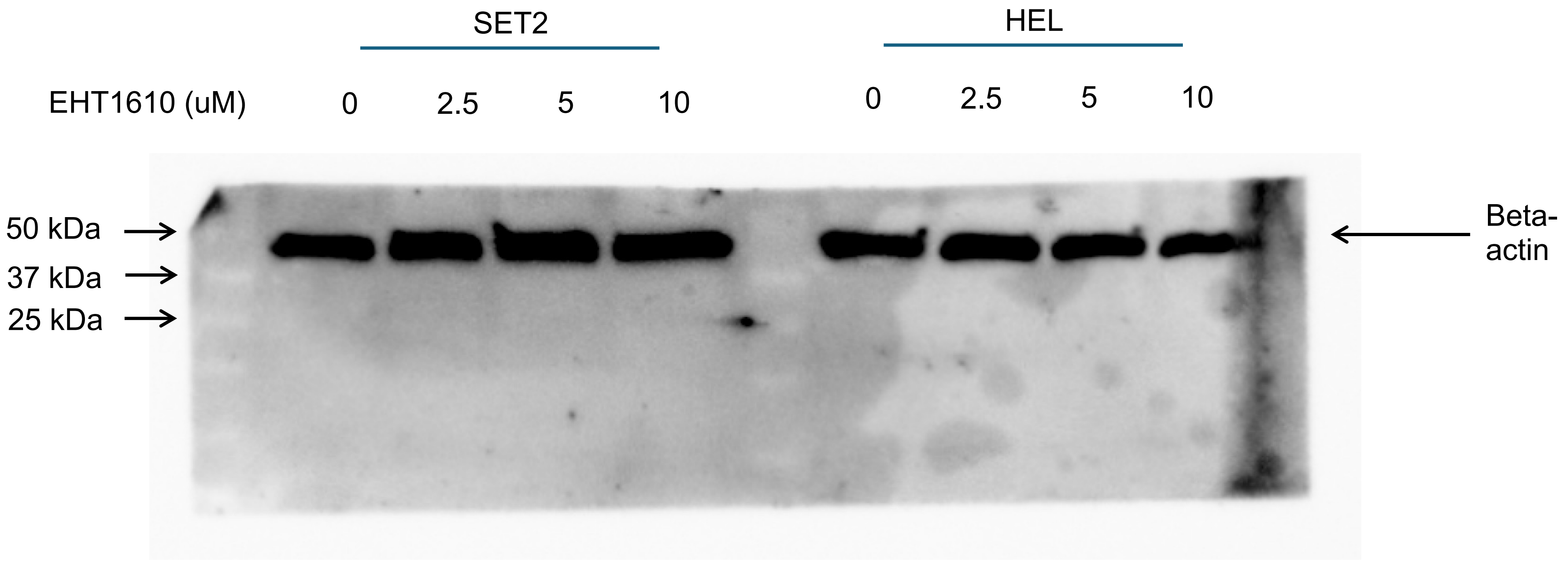

Supplement: Supplementary file 5 — Unprocessed western blots/gels. [file 41588_2025_2190_MOESM5_ESM.pdf]
